# Salicylic acid and methanol-induced changes in enhancing rapeseed resilience to drought stress conditions

**DOI:** 10.1038/s41598-026-43291-4

**Published:** 2026-05-05

**Authors:** Samaneh Safajoo, Ali Faramarzi, Jalil Ajalli, Mehrdad Abdi, Mehdi Oraei

**Affiliations:** 1 Department of Agronomy and Plant Breeding, Mi.C., Islamic Azad University, Miyaneh, Iran; 2 Department of Horticulture, Mi.C., Islamic Azad University, Miyaneh, Iran

**Keywords:** Antioxidant enzymes, Carbohydrates, Drought stress, Enzymatic/non-enzymatic activities, Oilseeds, Biochemistry, Physiology, Plant sciences

## Abstract

The present study aimed to investigate the effects of foliar applications of salicylic acid at 0 (control), 100, and 200 mg L^− 1^ and methanol at 0 (control), 10, and 20% (v/v) on morpho-physiological, biochemical, and yield-related attributes of rapeseed (*Brassica napus* L.) under drought stress conditions over two consecutive growing seasons. Drought stress was applied at three levels based on cumulative Class A pan evaporation, corresponding to well-watered at 70 mm, moderate drought at 140 mm, and severe drought at 210 mm. The experiment was conducted over two growing seasons using a split-factorial arrangement in a randomized complete block design with three replications. Drought stress significantly reduced grain yield, thousand-seed weight, and photosynthetic pigments, while increasing adaptive responses, such as proline, soluble sugars, and antioxidant enzyme activities. Foliar application of SA and methanol mitigated these negative effects. For instance, 200 mg L^− 1^ SA increased grain yield by 30.99 and 7.72% compared to control and 100 mg L^− 1^ SA under moderate drought, whereas 20% methanol enhanced grain yield by 31.62 and 15.93% compared to 10% methanol and control under severe drought stress. Chlorophyll a and relative water content also improved up to 61.99 and 66.28%, respectively, under optimal foliar treatments. Overall, although foliar application of 200 mg L^− 1^ SA was particularly effective under moderate drought stress, 20% methanol exhibited greater benefits under severe drought stress. The combination of SA and methanol at higher concentrations provided synergistic effects, enhancing physiological, biochemical, and yield-related traits under both irrigation regimes.

## Introduction

Agriculture has long served as a cornerstone of economic growth, structural transformation, and development, playing a crucial role in global food supply and raw material production^1–3^. Despite decades of advancement, agriculture remains the primary source of livelihood for millions, particularly in rural areas of developing nations, where poverty continues to be widespread^4,5^. As global population growth accelerates, demand for agricultural commodities is projected to rise by 35–56% by 2050^6,7^, underscoring the urgency of enhancing crop productivity and resilience. However, increasing climatic variability, particularly the intensification of drought events, has emerged as a major constraint on sustainable agricultural production, directly linking crop productivity to plant stress tolerance mechanisms^8,9^.

Oilseed crops are among the most valuable agricultural commodities, serving not only as major sources of edible oils but also as feedstocks for industrial and biofuel applications^10,11^. These crops are rich in essential nutrients, especially unsaturated fatty acids, play crucial roles in combating malnutrition, supporting human health, and generating employment opportunities, and contribute significantly to global food security and economic stability^12–14^. Rapeseed (*Brassica napus*) ranks as the second most important oilseed crop globally, possessing a balanced composition of omega-3, omega-6 and omega-9 fatty acids and accounting for approximately 12% of total vegetable oil production^15,16^. Beyond its culinary value, rapeseed is widely used in pharmaceutical, ornamental, and industrial sectors and serves as a protein-rich forage and a renewable source for biodiesel^15,17^. However, high percentages of erucic acid in the oil and glucosinolates in the meal restricted its consumption due to potential health concerns^17–19^. Despite its economic and nutritional importance, rapeseed is highly sensitive to environmental stresses, making yield stability strongly dependent on effective stress‑mitigation strategies^20,21^.

According to evidence, growth, development, and yield-related attributes are shaped by genetic, environmental, and management factors, and their complex interactions^22–24^. Rapeseed is particularly sensitive to environmental stressors throughout its growth stages, which can markedly impact both yield and oil-related attributes. Among these stressors, drought is one of the most detrimental, especially in arid and semi-arid regions. Drought stress can substantially reduce crop productivity, compromise food security, and threaten farmer livelihoods^25–27^. It adversely affects germination, seedling establishment, photosynthesis, nutrient uptake, seed development, and several morphological and physiological characteristics. Structural changes, such as alterations in stomatal density and conductance, epidermal and parenchymal tissue integrity, and elevated abscisic acid levels, are also common under water stress^28–32^. As such, developing effective strategies to mitigate drought stress is imperative. At the cellular level, drought stress disrupts redox homeostasis and accelerates the generation of reactive oxygen species (ROS), leading to oxidative damage to membranes, proteins, and photosynthetic machinery, thereby amplifying growth and yield losses^33,34^. Numerous studies have investigated compounds that enhance plant resilience under drought conditions. Among the most promising are plant growth regulators (PGRs), which help modulate physiological responses to stress^35^. Salicylic acid (SA), a key signaling molecule involved in systemic acquired resistance (SAR), plays a central role in stress mitigation by upregulating antioxidant defense mechanisms and improving drought tolerance by enhancing enzymatic/non-enzymatic antioxidant systems, stabilizing cell structures, and promoting plant growth under stress^36–39^. Furthermore, it inhibits ACC synthase, reducing ethylene synthesis and chlorophyll degradation^40,41^, and influences key physiological processes such as transpiration, stomatal regulation, membrane permeability, and seed performance^42–44^. In parallel, methanol has emerged as a potential carbon source that enhances photosynthesis and drought resistance^45,46^. Studies indicate that methanol can promote plant growth and productivity under drought conditions by stimulating metabolic and biosynthetic pathways^47,48^. Under drought stress conditions, methanol‑derived carbon may partially compensate for drought‑induced limitations in CO_2_ assimilation, thereby supporting photosynthetic activity and carbon metabolism (Foliar‑applied methanol can influence carbon metabolism and enhance photosynthetic activity under stressful conditions^49,50^. When used in combination, SA and methanol may exert synergistic effects by coordinately enhancing physiological performance and yield potential under drought stress. This potential synergy is hypothesized to arise from the complementary roles of salicylic acid in regulating stress signaling and redox homeostasis, and methanol in supporting carbon metabolism and photosynthetic efficiency, thereby collectively alleviating drought‑induced oxidative and metabolic constraints^45,51,52^.

Given these considerations, this study was designed to evaluate the effects of foliar application of varying concentrations of salicylic acid and methanol on selected growth, physiological, biochemical, and yield traits of rapeseed under different irrigation regimes in Dezful, Khuzestan Province, Iran. It was hypothesized that the combined application of salicylic acid and methanol would produce synergistic effects by integrating stress signaling regulation with metabolic compensation, thereby more effectively alleviating drought-induced damage than either treatment alone. Although previous studies have evaluated the effects of salicylic acid and methanol individually, limited information exists on their combined, concentration-dependent effects on morpho-physiological, biochemical, and yield traits of rapeseed under field conditions. This study addresses this research gap by investigating the potential synergistic interactions of these foliar treatments across two consecutive growing seasons, providing novel insights into how integrated physiological and biochemical adjustments can enhance drought tolerance. Emerging global analyses indicate that future vegetation dynamics may substantially alter terrestrial water loss, reinforcing the urgency of developing strategies to improve crop water-use efficiency under drought stress^53^. These findings highlight the urgency of developing agronomic strategies that enhance crop water-use efficiency under drought conditions, reinforcing the practical relevance of studies aimed at improving drought resilience in key crops such as rapeseed. As rapeseed holds strategic importance as an oilseed crop, the findings may offer valuable insights into managing drought stress and support the development of effective agronomic practices to enhance crop performance under adverse environmental conditions.

## Materials and methods

### Plant materials and experimental conditions

Seeds of rapeseed (*Brassica napus* L. var. Hayola 401) used in this study were obtained from farm-saved seeds harvested from previous cropping seasons in Dezful County, Khuzestan province, Iran. It should be emphasized that the investigated seeds were confirmed to be true-to-type and free from off-type contaminants, ensuring consistency and minimizing potential genetic variability in the experiment.

To assess the combined effects of SA and methanol foliar treatments on rapeseed performance under different water availability levels, a field study was carried out over two consecutive growing seasons (2018–2019 and 2019–2020) at a research farm located in Dezful County, southwestern Iran. The experimental layout followed a split-factorial arrangement within a randomized complete block design (RCBD) with three replications. Irrigation regimes were assigned to the main plots and defined based on cumulative evaporation from a Class A pan at three levels (70, 140, and 210 mm). Subplots consisted of factorial combinations of three SA concentrations (0, 100, and 200 mg L^− 1^) and three methanol levels (0, 10, and 20% v/v), applied as foliar sprays.

### Soil analysis and field preparation, and crop management

Before field establishment, representative soil samples were collected from the experimental area and analyzed for their physicochemical characteristics in a soil and water laboratory (Table 1). Then, the required nutrients were uniformly applied to all experimental plots based on the results of the soil analysis. Hence, nitrogen was applied as urea (46% N) in two top‑dressing applications, at stem elongation and early flowering stages, to support both vegetative growth and reproductive development. Phosphorus, potassium, and sulfur were applied as basal fertilizers before sowing, with P as triple superphosphate (46% P₂O₅), K as muriate of potash (60% K₂O), and S as ammonium sulfate (21% *N* + 21% S).

**Table 1:** Physico-chemical properties of the soil sample and nutritional recommendations.

Soil texture	Clay (%)	Silt (%)	Sand (%)	pH	EC (dS.m^− 1^)	Available nitrogen (%)	*P* (mg kg^− 1^)	K (mg kg^− 1^)
Loam-clay	34	35	31	7.1	0.99	41	6.9	108
Nutritional recommendations								
Urea 46% N (Kg ha^− 1^)		P (Kg ha^− 1^)			K (Kg ha^− 1^)		S (Kg ha^− 1^)	
100		60			70		25	

Following primary and secondary tillage and land preparation, experimental plots measuring 40 m^2^ (8 m × 5 m) were then established. Row spacing was 25 cm, and final plant density was adjusted to 40 plants m^− 2^. Initial irrigations were applied uniformly across all plots until the seedlings established. Thereafter, drought stress treatments were initiated and maintained throughout the growing season by regulating irrigation based on cumulative Class A pan evaporation thresholds (70, 140, and 210 mm), corresponding to well-watered, moderate drought, and severe drought stress conditions, respectively. Salicylic acid and methanol were employed at four critical points, including one pre-sowing treatment (seed priming) and three foliar applications at key growth stages, namely onset of the first drought stress (seedling establishment stage), stem elongation, and 50% silique formation. These points were chosen due to their high sensitivity to drought stress and their key roles in determining plant growth, photosynthetic capacity, and yield formation. Seed priming enhances early seedling vigor and stress resilience, the first drought onset targets early vegetative stress responses, stem elongation coincides with rapid biomass accumulation, and 50% silique formation is critical for reproductive success and yield determination. For seed priming, seeds were soaked in solutions containing the specified concentrations of SA and/or methanol for 12 h prior to sowing, then air-dried to their original moisture content before planting. For foliar applications at three key growth stages (onset of the first drought stress at seedling establishment, stem elongation, and 50% silique formation), solutions were prepared at the specified concentrations with 0.1% (v/v) Tween-20 as a surfactant. Foliar sprays were applied using a hand-held knapsack sprayer in the early morning to minimize evaporation and maximize uptake. The application volumes were adjusted proportionally to plot size, corresponding to 100 L ha^− 1^ for the seedling establishment stage and 400 L ha^− 1^ for the stem elongation and 50% silique formation stages. Each growth stage received a single foliar spray, with intervals between sprays determined by crop development stages as described above. This protocol ensured uniform coverage, reproducibility, and effective uptake of both SA and methanol under field conditions. This timing ensures that foliar treatments can effectively support osmotic adjustment, antioxidant defense, and carbon assimilation during the most vulnerable phases of rapeseed growth under the hot and arid conditions of Dezful, Iran.

### Measurement of morphological, biochemical, and yield traits

At the end of the experiment, plant height (PHt) was measured using a ruler. Yield-related traits, including silique length (SL), number of siliques per plant (SPP), grains per silique (GPS), 1000-grain weight (TGW), and grain yield (GY), were recorded based on the average of 10 phenotypically uniform plants per plot after excluding border effects. Biochemical analyses were also conducted at the 50% silique formation stage. The measured parameters included chlorophyll a (Chl a), chlorophyll b (Chl b), total chlorophyll (Chl T), carotenoids (CAR), catalase (CAT), ascorbate peroxidase (APX), and guaiacol peroxidase (GPX) activities, as well as soluble carbohydrate (SC) content and leaf proline (LP) content, using standard analytical procedures.

### Pigment assay (Chl a, b, total, and CAR)

Chl and CAR contents were determined using the method described by Arnon^54^ and Lichtenthaler^55^, respectively. Briefly, 0.5 g of fresh lamina from the leaf was homogenized in 80% acetone on ice using a mortar and pestle. The extract was filtered through Whatman filter paper into a volumetric flask. The residue was re-extracted with 80% acetone until the leaf pulp turned white. The final volume of the extract was adjusted to 10 mL with 80% acetone. The absorbance of the solution was read immediately at wavelengths of 645, 663, and 470 nm using a spectrophotometer (Jenway 6300). It should be noted that 80% aqueous acetone was employed as the Blank solution. Finally, the contents of Chl a, Chl b, T Chl, and CAR were calculated using standard equations as follows:

1 $$\mathrm{C}\mathrm{h}\mathrm{l}\mathrm{o}\mathrm{r}\mathrm{o}\mathrm{p}\mathrm{h}\mathrm{y}\mathrm{l}\mathrm{l}\mathrm{a}\left({\mathrm{m}\mathrm{g}\mathrm{g}}^{-1}\mathrm{F}\mathrm{W}\right)=\frac{[\left(12.7\times\mathrm{D}663\right)-\left(2.69\times\mathrm{D}645\right)]\times\mathrm{V}}{1000\times\mathrm{W}}$$

2 $$\mathrm{C}\mathrm{h}\mathrm{l}\mathrm{o}\mathrm{r}\mathrm{o}\mathrm{p}\mathrm{h}\mathrm{y}\mathrm{l}\mathrm{l}\mathrm{b}\left({\mathrm{m}\mathrm{g}\mathrm{g}}^{-1}\mathrm{F}\mathrm{W}\right)=\frac{[\left(22.9\times\mathrm{D}645\right)-\left(4.93\times\mathrm{D}663\right)]\times\mathrm{V}}{1000\times\mathrm{W}}$$

3 $$\mathrm{T}\mathrm{o}\mathrm{t}\mathrm{a}\mathrm{l}\mathrm{C}\mathrm{h}\mathrm{l}\mathrm{o}\mathrm{r}\mathrm{o}\mathrm{p}\mathrm{h}\mathrm{y}\mathrm{l}\mathrm{l}\left({\mathrm{m}\mathrm{g}\mathrm{g}}^{-1}\mathrm{F}\mathrm{W}\right)=\frac{[\left(20.2\times\mathrm{D}645\right)-\left(8.02\times\mathrm{D}663\right)]\times\mathrm{V}}{1000\times\mathrm{W}}$$

4 $$\mathrm{C}\mathrm{a}\mathrm{r}\mathrm{o}\mathrm{t}\mathrm{e}\mathrm{n}\mathrm{o}\mathrm{i}\mathrm{d}\mathrm{s}\left({\mathrm{m}\mathrm{g}\mathrm{g}}^{-1}\mathrm{F}\mathrm{W}\right)=\frac{\left[\left(1000\times\mathrm{D}470)-(1.82\times\mathrm{C}\mathrm{h}\mathrm{l}.\mathrm{a})-(85.02\times\mathrm{C}\mathrm{h}\mathrm{l}.\mathrm{b}\right)\right]}{198}$$

### Leaf proline (LP) content

Proline content was quantified using the method presented by Bates et al.^56^ with a slight modification based on the reaction between free proline and ninhydrin under acidic conditions at 100 °C, producing a red-colored complex extractable in toluene. In this assay, 0.5 g of fresh leaf tissue was homogenized in 10 mL of 3% sulfosalicylic acid and filtered with Whatman filter paper No.1. Then, 2 mL of the filtered extract was mixed with 2 mL of acid-ninhydrin reagent and 2 mL of glacial acetic acid. The obtained mixture was heated in a water bath at 100 °C for 60 min and then cooled on an ice-water bath for 30 min. Four mL of toluene were added, vortexed for 15–20 s, and allowed to separate into two phases at room temperature. The upper (colored) toluene layer was collected, and its absorbance was measured at 520 nm using a spectrophotometer. Eventually, the proline concentration was estimated based on the standard curve at concentrations of 0, 50, 100, 200, and 250 µM (Eq. 5).

5 $$\mathrm{P}\mathrm{r}\mathrm{o}\mathrm{l}\mathrm{i}\mathrm{n}\mathrm{e}\mathrm{c}\mathrm{o}\mathrm{n}\mathrm{c}\mathrm{e}\mathrm{n}\mathrm{t}\mathrm{r}\mathrm{a}\mathrm{t}\mathrm{i}\mathrm{o}\mathrm{n}\left({\upmu}\mathrm{M}{\mathrm{g}}^{-1}\mathrm{F}\mathrm{W}\right)=\frac{\left[\frac{\left({{\upmu}\mathrm{g}.\mathrm{m}\mathrm{l}}^{-1}\mathrm{p}\mathrm{r}\mathrm{o}\mathrm{l}\mathrm{i}\mathrm{n}\mathrm{e}\times\mathrm{m}\mathrm{l}\mathrm{t}\mathrm{o}\mathrm{l}\mathrm{u}\mathrm{e}\mathrm{n}\mathrm{e}\right)}{115.5{{\upmu}\mathrm{g}.{\upmu}\mathrm{m}\mathrm{o}\mathrm{l}\mathrm{e}}^{-1}}\right]}{\left[\frac{\left(\mathrm{g}\mathrm{s}\mathrm{a}\mathrm{m}\mathrm{p}\mathrm{l}\mathrm{e}\right)}{5}\right]}$$

### Catalase (CAT) activity

CAT activity was determined using a 50 mM potassium phosphate buffer containing 1 mM EDTA and 2% PVPP at 4 °C, following the method described by Chaoui et al.^57^. The decomposition of H₂O₂ was monitored by measuring the decrease in absorbance at 240 nm from the reaction mixture, which consisted of 25 mM phosphate buffer (pH 7.0), 10 mM H₂O₂, and the enzyme extract. Accordingly, One g of fresh tissue was first ground in a mortar with 3 mL of 50 mM phosphate buffer (pH 7.2) containing 1 mM EDTA, 1 mM PMSF, and 1% PVP for enzyme extraction. The homogenate was centrifuged at 14,000 × g for 15 min at 4 °C, and the supernatant was used for enzymatic measurements. Eventually, enzyme activity was calculated using the Beer-Lambert law as follows:

6 $$ A = \varepsilon bc $$

Where A, ε, b, and c represent the recorded absorbance, extinction coefficient (= 0.036 mM^− 1^ cm^− 1^), cuvette diameter, and H_2_O_2_ concentration, respectively.

Also, total protein content in all enzyme extracts was determined using the method described by Bradford^58^ with bovine serum albumin as a standard. Enzyme activities were subsequently expressed per mg of protein.

### Ascorbate peroxidase (APX) activity

APX activity was measured following the method of Nakano and Asada^59^. For the assay, 1 g of fresh leaf tissue was homogenized in 3 mL of 50 mM phosphate buffer (pH 7.0) containing 1 mM EDTA, 1 mM PMSF, and 1% PVP, and centrifuged at 14,000 × g for 15 min at 4 °C. The enzyme activity was determined in a 2 mL reaction mixture containing 250 mM phosphate buffer (pH 7.0), 0.1 mM EDTA, 0.5 mM ascorbate, and 1.2 mM H_2_O_2_, and was calculated by monitoring the decrease in absorbance at 290 nm using the previously defined Beer–Lambert law with an extinction coefficient of 2.8 mM^− 1^ cm^− 1^. Results were expressed per unit of total protein content.

### Guaiacol peroxidase (GPX) activity

GPX activity was assayed according to Chance and Maehly^60^ at 25 °C. The reaction mixture (3 mL total) contained 50 mM monosodium phosphate buffer (pH = 7), 10 µL of 30% H₂O₂, 3 µL of 200 mM guaiacol, and 50 µL of enzyme extract. The reaction was initiated with guaiacol, and the absorbance was immediately measured at 470 nm for 3 min at 20-second intervals. Finally, enzymatic activity was calculated based on the formation rate of tetraguaiacol and expressed in units per mg of protein.

### Soluble carbohydrates (SC) content

SC were determined based on the method of Yemm and Willis^61^. Ground plant samples were extracted in 95% ethanol and centrifuged at 4 °C. A freshly prepared anthrone reagent (150 mg anthrone in 100 mL of 72% sulfuric acid) was added to the extract. The tubes were heated in a water bath for 10 min, cooled, and absorbance was read at 625 nm using a spectrophotometer (Cary 100 Conc, USA).

### Relative water content (RWC)

To determine the Relative water content (RWC), 0.5 g of fully expanded leaves was weighed immediately after excision to determine the fresh weight (FW), ensuring no loss of water content. The samples were then floated in distilled water under dark conditions at 4 °C for 24 h to achieve full turgidity and obtain the turgid weight (TW). Afterward, leaves were oven-dried at 70 °C for 24 h to determine dry weight (DW). Furthermore, RWC was calculated using the following formula:

7 $$RWC\left(\%\right)=\left(\frac{FW-DW}{TW-DW}\right)\times100$$

### Oil percentage (OP) and oil yield (OY)

The OP was determined using Soxhlet extraction from the harvested grains of each plot. OY was also calculated by multiplying GY by OP.

### Statistical analysis

The data were analyzed using SAS software (version 9.4). Mean comparisons were performed using Fisher’s LSD test at *p* < 0.05. Graphs were generated using Microsoft Excel. Prior to analysis of variance, Bartlett’s test was applied to assess the homogeneity of variances.

## Results

At the outset, it should be noted that because of the heterogeneity of variance based on Bartlett’s test, the data from the experimental treatments were analyzed and reported separately for each year as follows:

### Morpho-physiological traits

#### Plant height (PHt) and silique length (SL)

Analysis of variance (Table 2) showed that PHt was significantly influenced by all main treatments, including irrigation regimes (IR), salicylic acid (SA) foliar application, and methanol levels (*p < 0.01*) in the first year of the experiment. In addition, the effects of IR, SA concentrations, and methanol levels on stem length (SL) were significant at the 1%, 5%, and 5% probability levels, respectively, during the first year. However, all main treatments had a significant effect on SL at *p* < 0.01 in the second year.

**Table 2:** Analysis of variance of the effects of irrigation regimes, SA, and methanol on some morpho-physiological traits of rapeseed.

Source of variation	df	PHt		SL		RWC	
		First year data	Second year data	First year data	Second year data	First year data	Second year data
Block ^O^	2	1.36 ns	5.35 ns	0.01 ns	0.03 ns	9.35 ns	2.61 ns
irrigation regimes (IR)	2	1983.37**	3791.08**	32.21**	56.35**	8255.05**	4732.9**
Error a (Ea)^●^	4	54.64	88.28	0.12	0.16	18.66	65.12
SA concentrations	2	410.63**	1066.55**	1.93*	1.79**	859.6**	897.49**
Methanol levels	2	255.34**	494.04**	1.11 *	1.84**	149.86 ns	347.86**
IR × SA	4	15.64 ns	113.88 ns	0.18 ns	0.27 ns	50.91 ns	50.52 ns
IR × Methanol	4	2.58 ns	5.87 ns	0.09 ns	0.16 ns	6.57 ns	1.24 ns
SA × Methanol	4	12.93 ns	39.19 ns	0.19 ns	0.19 ns	6.57 ns	21.58 ns
IR × SA × Methanol	8	5.41 ns	7.05 ns	0.1 ns	0.14 ns	11.55 ns	17.74 ns
Error b (Eb)^■^	48	57.64	68.74	0.42	0.22	183.66	569.75
CV (%)		7.07	8.92	11.64	11.23	12.21	11.41

ns, *, and **; non-significant, significant at *p* < 0.05 and significant at *p* < 0.01, respectively.

O Block: experimental replication.

● Ea: experimental error for the main-plot factor (Block × Treatment A).

■ Eb: experimental error for the subplot factor (Block × Treatment A × Treatment B).

Irrigation regimes (IR) represent cumulative evaporation levels of 70, 140, and 210 mm during the growing season. Foliar applications included SA at 0 (control), 100, and 200 mg^− 1^, and methanol at 0 (control), 10, and 20% (v/v).

The mean comparisons related to the effects of main treatments on PHt and SL (Table 3) showed that the highest PHt (111.84 cm) was recorded under irrigation after 70 mm of pan evaporation. However, it was not significantly different from the PHt observed under 140 mm evaporation (106.75 cm). The lowest PHt (95.16 cm) was observed under the 210 mm evaporation treatment. Regarding the effect of SA application on rapeseed plants in the first year of the experiment, the lowest and highest PHt values (100.43 and 108.49 cm, respectively) were recorded in the control (no SA) and 200 mg L^− 1^ SA treatments. Similarly, methanol treatment results showed that the lowest and highest PHt values (101.19 and 107.09 cm) occurred in the control and 20% methanol treatments, respectively.

**Table 3:** Mean comparisons (mean squares) effects of IR, SA, and methanol levels on some traits of rapeseed.

Source of variation	PHt (cm)		SL (cm)		CAT (mM H_2_O_2_ mg^− 1^ protein min^− 1^)	GPX (U mg^− 1^ protein min^− 1^)		APX (U mg^− 1^ protein min^− 1^)		SPP (Numbers)	
	First year data	Second year data	First year data	Second year data	Second year data	First year data	Second year data	First year data	Second year data	First year data	Second year data
IR (mm of evaporation)											
70	111.84a	102.68a	6.83 a	5.87a	0.390b	0.321c	0.643c	2.24c	3.36c	66.16a	51.93a
140	106.75a	96.4a	5.4 b	3.68b	0.502a	0.448b	0.724b	3.29b	4.44b	49.23b	34.47b
210	95.16b	79.75b	4.65 b	3.13c	0.543a	0.491a	0.828a	3.38a	5.35a	40.54c	40.54c
LSD	5.73	7.09	0.26	0.3	0.034	0.018	0.110	0.20	0.17		
SA (mg L^− 1^)											
0 (control)	100.63b	86.52c	5.45 b	3.94b	0.509a	0.449a	0.856a	3.23a	5.38a	48.04b	31.19c
100	104.62ab	93.22b	5.51 b	4.28a	0.470b	0.411b	0.700b	3.02b	4.11b	51.31b	34.79b
200	108.49a	99.09a	5.92 a	4.46a	0.448c	0.402b	0.693c	2.96c	3.61c	56.58a	41.26a
Methanol levels (v/v)											
0 (control)	101.19b	88.14b	5.41 b	3.97c	0.495a	0.448a	0.788a	3.30a	4.82a	48.12b	30.43c
10	105.47a	94.36a	5.66 ab	4.23b	0.474b	0.415b	0.712b	3.02b	4.20b	51.04b	35.84b
20	107.09a	96.33a	5.81 a	4.49a	0.457b	0.398b	0.696b	2.98c	4.09b	56.77a	40.97a
LSD	4.04	4.53	0.35	0.26	0.020	0.019	0.045	0.14	0.30		

Same superscript letters assigned to treatment means in a column indicate non-significant differences from each other at *p < 0.05*.

Irrigation regimes (IR) represent cumulative evaporation levels of 70, 140, and 210 mm during the growing season. Foliar applications included SA at 0 (control), 100, and 200 mg^− 1^, and methanol at 0 (control), 10, and 20% (v/v).

In the second year (Table 3), PHt varied across IR, SA, and methanol treatments. For IR treatment, the lowest and highest PHt were observed under the 210 mm and 70 mm evaporation treatments, measuring 79.75 cm and 102.68 cm, respectively. Under the SA application, PHt ranged from 86.52 cm (200 mg L^− 1^ SA) to 99.09 cm (control), and for methanol levels, it ranged from 77.14 cm (control) to 96.33 cm (20% methanol). Also, ANOVA results (Table 2) indicated that SL was significantly affected by all treatments. Mean comparisons showed that the longest SL in both years (6.83 and 5.87 cm) occurred under the 70 mm evaporation treatment. In contrast, the shortest SL (4.56 and 3.13 cm) were recorded under the 210 mm evaporation treatment. Regarding SA, maximum and minimum SL values were 5.92 cm and 5.45 cm in the first year and 4.46 cm and 3.94 cm in the second year, corresponding to 200 mg L^− 1^ SA and control (no SA) treatments. Similarly, the longest SL in both years was obtained under 20% methanol (Table 3).

#### Relative water content (RWC)

According to the analysis of variance (Table 2), RWC was significantly affected by IR and SA foliar application during the first year (*p < 0.01*). In the second year, all three main treatments, IR, SA application, and methanol concentrations, had a significant effect on RWC (*p < 0.01*). However, no significant interaction effects among the treatments were observed in either year.

**Fig. 1:**
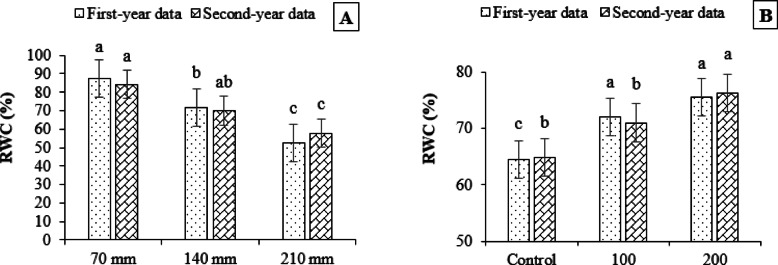
Effects of (**a**) IR and (**b**) SA concentrations on RWC. Same superscript letters assigned to treatment means in a column indicate non-significant differences from each other at *p* < 0.05. ***Foliar applications included SA at 0 (control), 100, and 200 mg L^− 1^.

Mean comparisons for the first year revealed that the lowest and highest RWC values were obtained under the 210 mm and 70 mm pan evaporation treatments, amounting to 52.7% and 87.63%, respectively (Fig. 1a). Likewise, foliar application of SA had a significant effect, with the lowest and highest RWC values recorded in the control and 200 mg L⁻¹ SA treatments (64.48% and 75.52%, respectively; Fig. 1b). A similar trend was observed in the second year. The highest and lowest RWC values (84.3% and 57.85%) were again associated with the 70 mm and 210 mm irrigation treatments, respectively. Application of the 70 mm treatment significantly improved RWC by 20.51% and 45.72% compared to the 140 mm and 210 mm regimes (Fig. 1a). SA treatment also had a notable effect on RWC, with the maximum and minimum values (76.3% and 64.78%, respectively) corresponding to 200 mg L^− 1^ SA and the control (Fig. 1b).

**Fig. 2:**
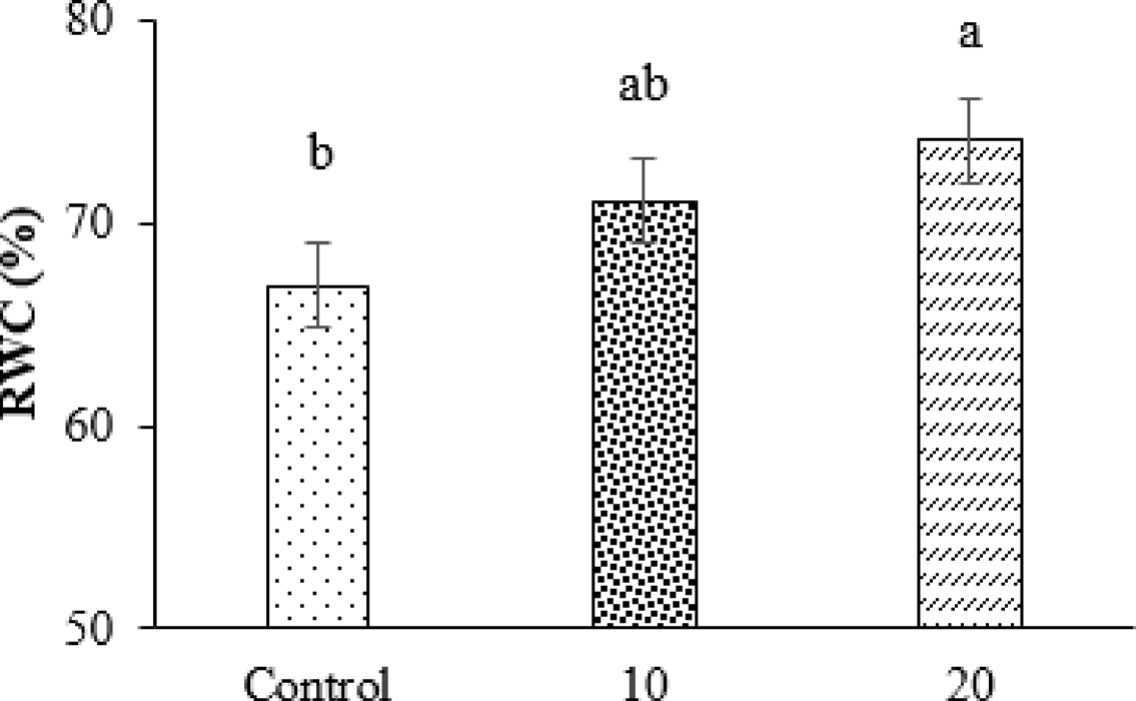
Effects of methanol levels on RWC (Adapted to the second-year data). Same superscript letters assigned to treatment means in a column indicate non-significant differences from each other at *p* < 0.05. *** Foliar applications included methanol at 0 (control), 10, and 20% (v/v).

Methanol treatments during the second year demonstrated that the highest RWC (74.07%) was obtained under the 20% methanol treatment, although the difference was not statistically significant compared to the 10% methanol treatment (71.07%). The lowest RWC under methanol treatments (66.93%) was recorded in the control group (Fig. 2).

### Biochemical traits

#### Chlorophyll a (Chl a), chlorophyll b (Chl b), total chlorophyll (Chl T), and carotenoids (CAR)

The ANOVA results for the first- and second-year data (Table 4) indicated significant effects of IR, SA concentrations, and methanol levels on photosynthetic pigments. Chl a content was significantly influenced by all three main treatments in both years (*p < 0.01*). Chl b was affected by IR and the IR × methanol interaction (*p < 0.05*) and by SA concentrations (*p < 0.01*) in the first year, whereas in the second year, it was influenced by IR, SA, and the interactions IR × methanol and SA × methanol (*p < 0.01*). Chl T showed significant variation under IR, SA, and methanol in the first year (*p < 0.01*), and under IR, SA, methanol, and the SA × methanol interaction in the second year (*p < 0.01*). Carotenoid content was significantly affected by IR, SA, and methanol as well as IR × SA in the first year (*p < 0.01*), and by IR (*p < 0.05*), SA, SA × methanol, and the three-way interaction IR × SA × methanol in the second year (*p < 0.01*).

**Table 4:** Analysis of variance (mean square) of the effects of irrigation regime, SA concentrations, and methanol levels on some biochemical traits of rapeseed.

Source of variation	df	First year data				Second year data			
		Chl a	Chl b	Chl T	CAR	Chl a	Chl b	Chl T	CAR
Block ^O^	2	0.02 ns	0.09 ns	0.24 ns	0.07 ns	0.32 ns	0.17*	0.27 ns	0.65 ns
Irrigation regimes (IR)	2	12.83**	0.16*	15.66**	50.76**	22.95**	0.66**	15.52**	1.91*
Error a (Ea)^●^	4	0.02	0.06	0.02	0.17	0.04	0.06	0.15	0.99
SA concentrations	2	7.86**	0.94**	14.13**	5.04**	13.87**	4.79**	34.92**	13.86**
Methanol levels	2	3.39**	0.02ns	3.36**	2.38**	6.72**	0.07ns	6.19**	0.44ns
IR × SA	4	0.06ns	0.07ns	0.08ns	1.02**	0.18ns	0.09ns	0.29ns	0.24ns
IR × Methanol	4	0.03ns	0.14*	0.25ns	0.02ns	0.34ns	0.63**	0.09ns	0.51ns
SA × Methanol	4	0.12ns	0.04ns	0.23ns	0.21ns	0.2ns	0.21**	0.79**	1.54**
IR × SA × Methanol	8	0.13ns	0.01ns	0.18ns	0.31ns	0.17ns	0.08ns	0.19ns	1.19**
Error b (Eb)^■^	48	0.19	0.04	0.34	0.21	0.22	0.05	0.28	0.56
CV (%)		15.43	26.38	15.95	19.66	16.55	13.81	11.87	21.27

ns, *, and **; non-significant, significant at *p* < 0.05 and significant at *p* < 0.01, respectively.

O Block: experimental replication.

● Ea: experimental error for the main-plot factor (Block × Treatment A).

■ Eb: experimental error for the subplot factor (Block × Treatment A × Treatment B).

Irrigation regimes (IR) represent cumulative evaporation levels of 70, 140, and 210 mm during the growing season. Foliar applications included SA at 0 (control), 100, and 200 mg^− 1^, and methanol at 0 (control), 10, and 20% (v/v).

**Fig. 3:**
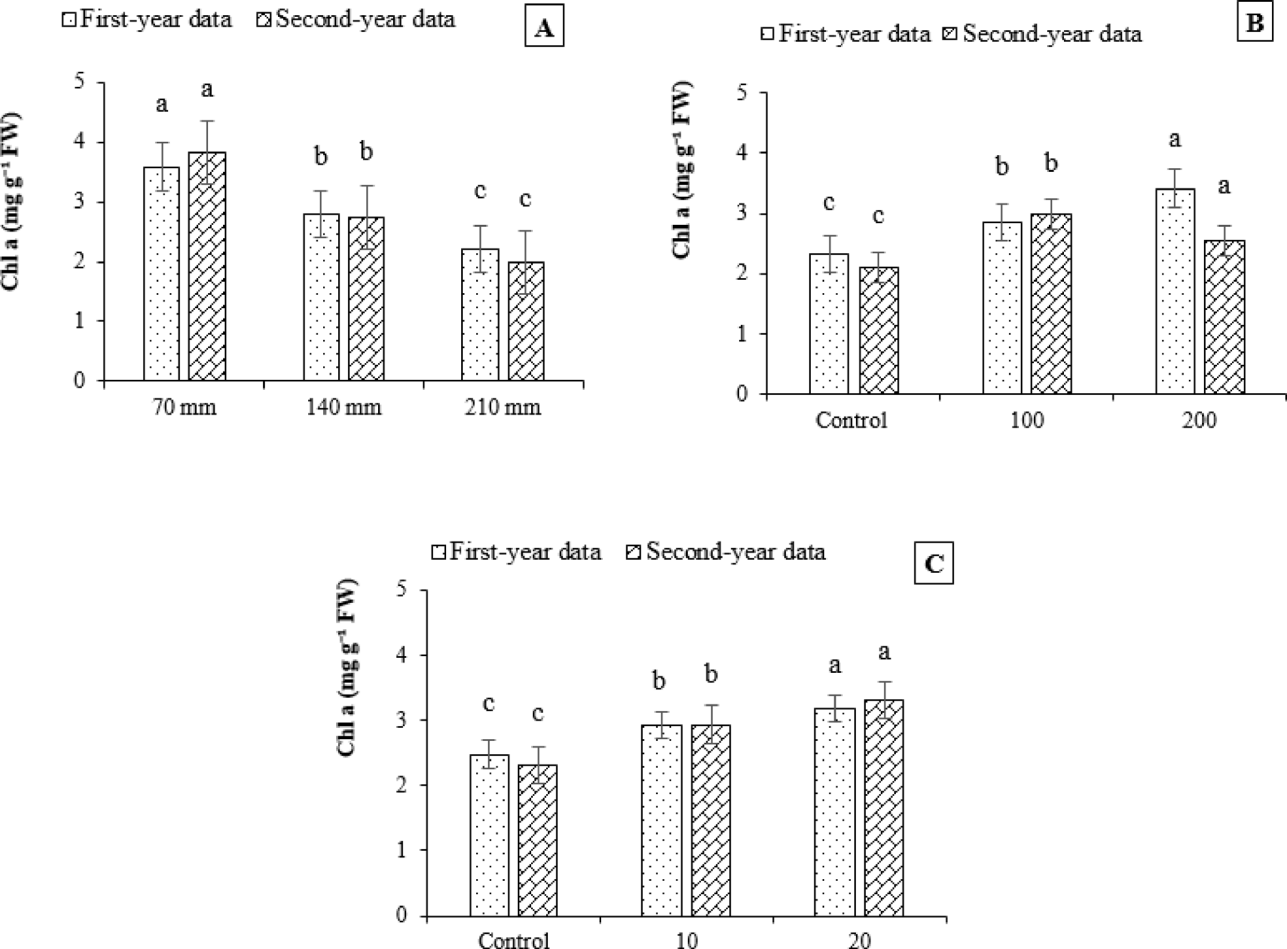
Effects of (**a**) IR, (**b**) SA concentrations, and (**c**) methanol levels on the content of Chl a. Same superscript letters assigned to treatment means in a column indicate non-significant differences from each other at *p* < 0.05. *** Foliar applications included SA at 0 (control), 100, and 200 mg L^− 1^ and methanol at 0 (control), 10, and 20% (v/v).

Mean comparisons of the IR on photosynthetic pigments during the first year (Fig. 3a) demonstrated that the highest and lowest chlorophyll a contents were observed in plants treated with 70 mm and 210 mm of evaporation, respectively (3.58 and 2.21 mg g^− 1^ FW). Additionally, considering the significant effects of different SA concentrations on the Chl a content in the first year, the highest and lowest significant values (3.41 and 2.33 mg g^− 1^ FW) were recorded for the 200 mg L^− 1^ SA treatment and the control, respectively. Accordingly, the application of 200 mg L^− 1^ SA on rapeseed plants resulted in significant increases of 46.35% and 19.65% in the Chl a content compared to the control and 100 mg L^− 1^ SA treatments, respectively (Fig. 3b; first-year data). Moreover, the effects of different methanol levels in the first year indicated that chlorophyll a content under control, 10%, and 20% methanol treatments were 2.48, 2.92, and 3.19 mg g^− 1^ FW, respectively. The results demonstrate significant increases of 28.63% and 9.25% in chlorophyll a content in plants treated with 20% methanol compared to the control and 10% methanol treatments, respectively (Fig. 3c; first-year data).

Based on the second-year data, it was observed that all three main treatments, IR, SA concentrations, and methanol levels, had significant effects on the Chl a content (see ANOVA shown in Table 4). Mean comparisons of IR on Chl a (see Fig. 7a) revealed that plants subjected to the 70 mm of evaporation exhibited the highest Chl a content, averaging 3.82 mg g^− 1^ FW. Significant effects of different SA concentrations on the Chl a content were also observed during the second year. The highest and lowest significant chlorophyll a values (2.98 and 2.12 mg g^− 1^ FW) were recorded for the 100 mg L^− 1^ SA treatment and the control (no SA), respectively. Further, the effects of foliar methanol application on chlorophyll a content in rapeseed during the second year (Fig. 3c) demonstrated that the highest chlorophyll a content (3.31 mg g^− 1^ FW) was obtained under the 20% methanol treatment. This represented significant increases compared to chlorophyll a content under the control (2.32 mg g^− 1^ FW) and 10% methanol treatments (2.94 mg g^− 1^ FW).

**Fig. 4:**
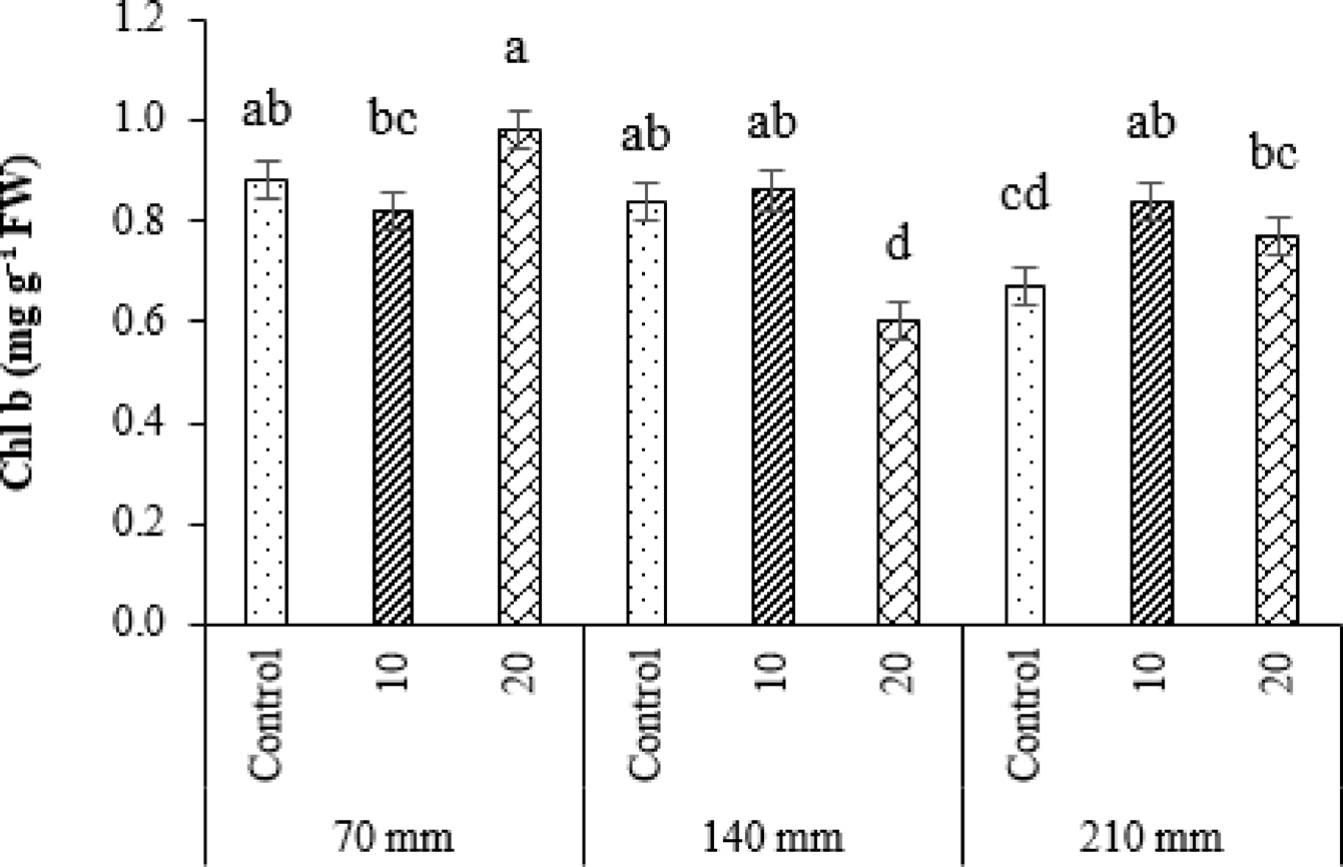
The interaction of IR × Methanol levels on the content of Chl b (Adapted to the first-year data). Same superscript letters assigned to treatment means in a column indicate non-significant differences from each other at *p* < 0.05. *** Foliar applications included methanol at 0 (control), 10, and 20% (v/v).

Regarding the significant interaction of IR × methanol levels on Chl b during the first year of the experiment (Table 4), mean comparisons (Fig. 4) revealed that the lowest and highest chlorophyll b contents (0.67 and 0.98 mg g⁻¹ FW) were observed under the interactions of 210 mm of evaporation × no methanol application and 70 mm of evaporation × 20% methanol, respectively. This suggests that methanol application mitigates chlorophyll degradation under moderate drought stress.

**Fig. 5:**
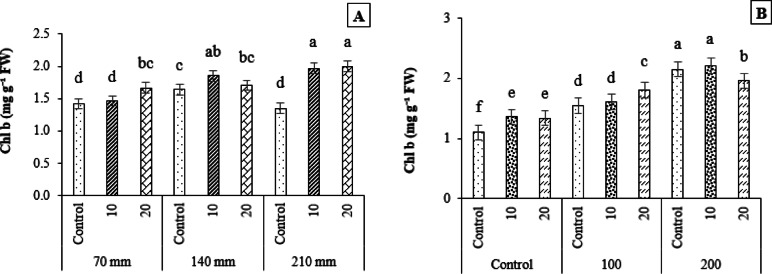
Interactions of (**a**) IR × Methanol and (**b**) SA concentrations × Methanol on the content of Chl b (Adapted to the second-year data). Same superscript letters assigned to treatment means in a column indicate non-significant differences from each other at *p* < 0.05. *** Foliar applications included SA at 0 (control), 100, and 200 mg L^− 1^ and methanol at 0 (control), 10, and 20% (v/v).

Regarding Chl b content in the second year (Fig. 5), the highest values were observed under the IR × methanol interactions of 210 mm × 20% methanol, 210 mm × 10% methanol, and 140 mm × 10% methanol, with mean values of 2.00, 1.97, and 1.85 mg g^− 1^ FW, respectively, without significant differences among them. The lowest Chl b contents were recorded under 210 mm × no methanol, 70 mm × no methanol, and 210 mm × 10% methanol, with means of 1.35, 1.42, and 1.46 mg g^− 1^ FW, respectively, although these differences were not statistically significant (Fig. 5a). For the SA × methanol interaction, the highest Chl b content (2.21 mg g^− 1^ FW) was observed under 200 mg L^− 1^ SA × 10% methanol, which did not differ significantly from 200 mg L^− 1^ SA × 20% methanol (1.96 mg g^− 1^ FW). The lowest significant Chl b content in the second year (1.10 mg g^− 1^ FW) was recorded in plants receiving no SA and no methanol (Fig. 5b). Chl b content was markedly higher when 210 mm IR was combined with 20% methanol, indicating a protective effect of methanol under moderate water stress conditions, likely through enhanced light-harvesting efficiency and reduced photodamage.

**Fig. 6:**
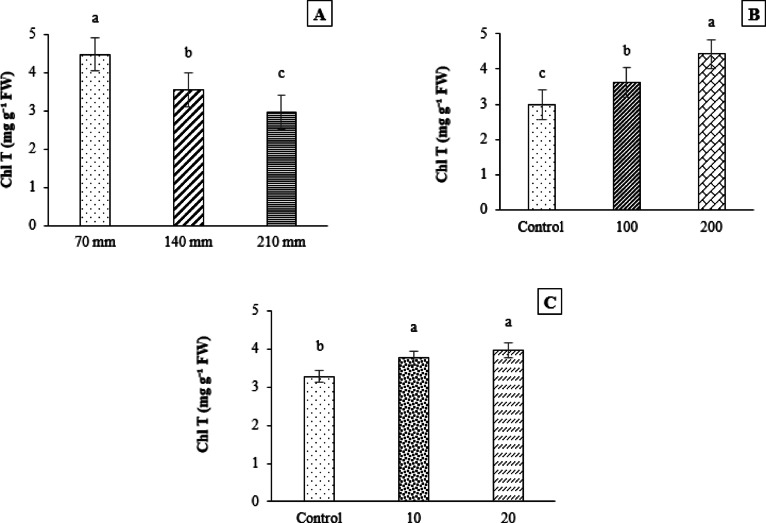
Effects of (**a**) IR, (**b**) SA concentrations, and (**c**) methanol levels on the content of Chl T (Adapted to the first-year data). Same superscript letters assigned to treatment means in a column indicate non-significant differences from each other at *p* < 0.05. *** Foliar applications included SA at 0 (control), 100, and 200 mg L^− 1^ and methanol at 0 (control), 10, and 20% (v/v).

The effects of IR on the Chl T content during the first year of the study (Fig. 6) demonstrated that the highest significant total chlorophyll content (48.4 mg g^− 1^ FW) was recorded under the 70 mm of evaporation treatment. In other words, plants subjected to the 70 mm of evaporation exhibited total chlorophyll contents that were 25.84% and 84.50% higher than those of plants treated with 140 mm and 210 mm of evaporation regimes, respectively (Fig. 6a). Furthermore, it was observed that the increase in total chlorophyll content was dependent on SA concentration, where foliar application of 200 mg L^− 1^ SA (42.4 mg g^− 1^ FW) caused significant increases of 48.32% and 22.44% compared to the control treatment (9.8 mg g^− 1^ FW) and the 100 mg L^− 1^ SA treatment (36.1 mg g^− 1^ FW), respectively (Fig. 6b). Application of different methanol levels also exerted significant and concentration-dependent effects on Chl T, with the highest total chlorophyll content (39.7 mg g⁻¹ FW) observed when rapeseed plants were treated with 20% methanol (Fig. 6c; First-year data).

**Fig. 7:**
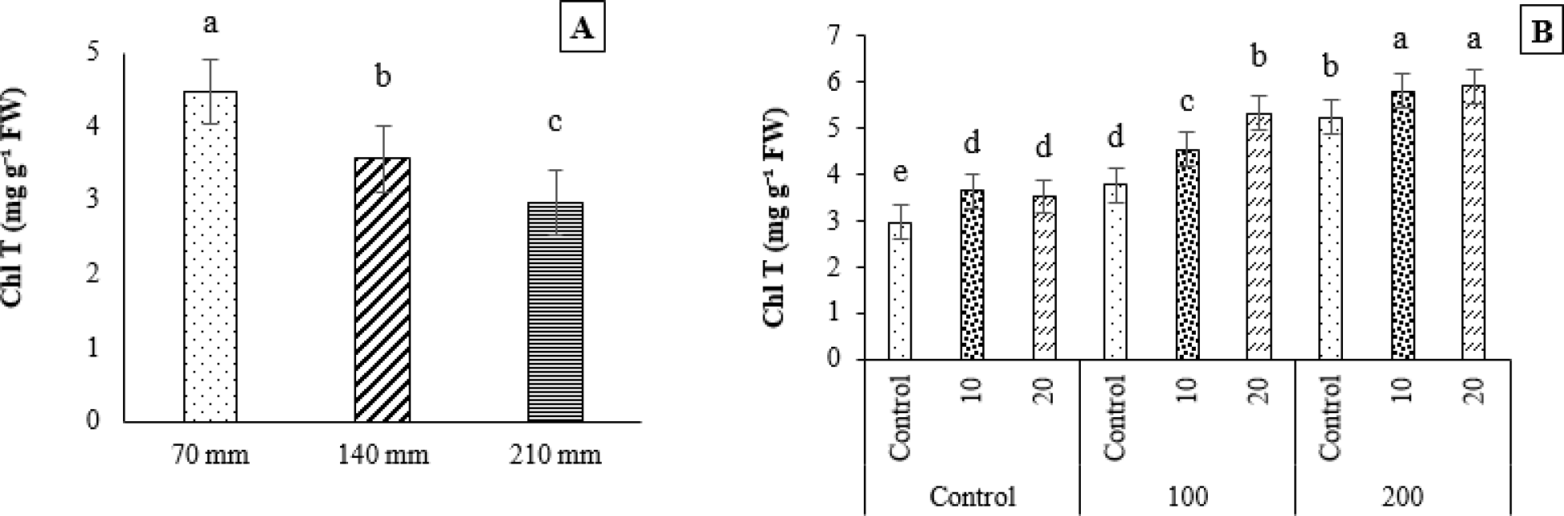
Effects of (**a**) IR and (**b**) SA concentrations × Methanol on the content of Chl T (Adapted to the second-year data). Same superscript letters assigned to treatment means in a column indicate non-significant differences from each other at *p* < 0.05. *** Foliar applications included SA at 0 (control), 100, and 200 mg L^− 1^ and methanol at 0 (control), 10, and 20% (v/v).

Since all main treatments of IR, SA concentration, and methanol levels, as well as the interaction of SA × methanol significantly affected the content of Chl T in rapeseed plants treated during the second year of the experiment (Fig. 7), the effects of the irrigation regime (as the main plot treatment) and the two-way interaction between SA concentration and methanol levels on this trait are further discussed. Irrigation regime effects on Chl T content during the second year showed that plants under the 70, 140, and 210 mm of evaporation contained 4.48, 3.35, and 2.97 mg g^− 1^ FW, respectively (Fig. 7a). Mean comparisons related to the interaction of SA concentrations × methanol levels (Fig. 7b; the second-year data) indicated that mean comparisons revealed that the highest Chl T content (5.92 mg g^− 1^ FW) was obtained under the interaction of 200 mg L^− 1^ SA × 20% methanol, which did not significantly differ from the under the 200 mg L^− 1^ SA × 10% methanol interaction (5.81 mg g⁻¹ FW). In contrast, the lowest Chl T content (2.97 mg g^−^¹ FW) was recorded for plants under the interaction of no SA × no methanol. These results suggest that the combined foliar application of SA and methanol can synergistically enhance total chlorophyll content under water-limited conditions, likely by improving chloroplast stability and reducing drought-induced oxidative damage, thereby supporting higher photosynthetic capacity.

**Fig. 8:**
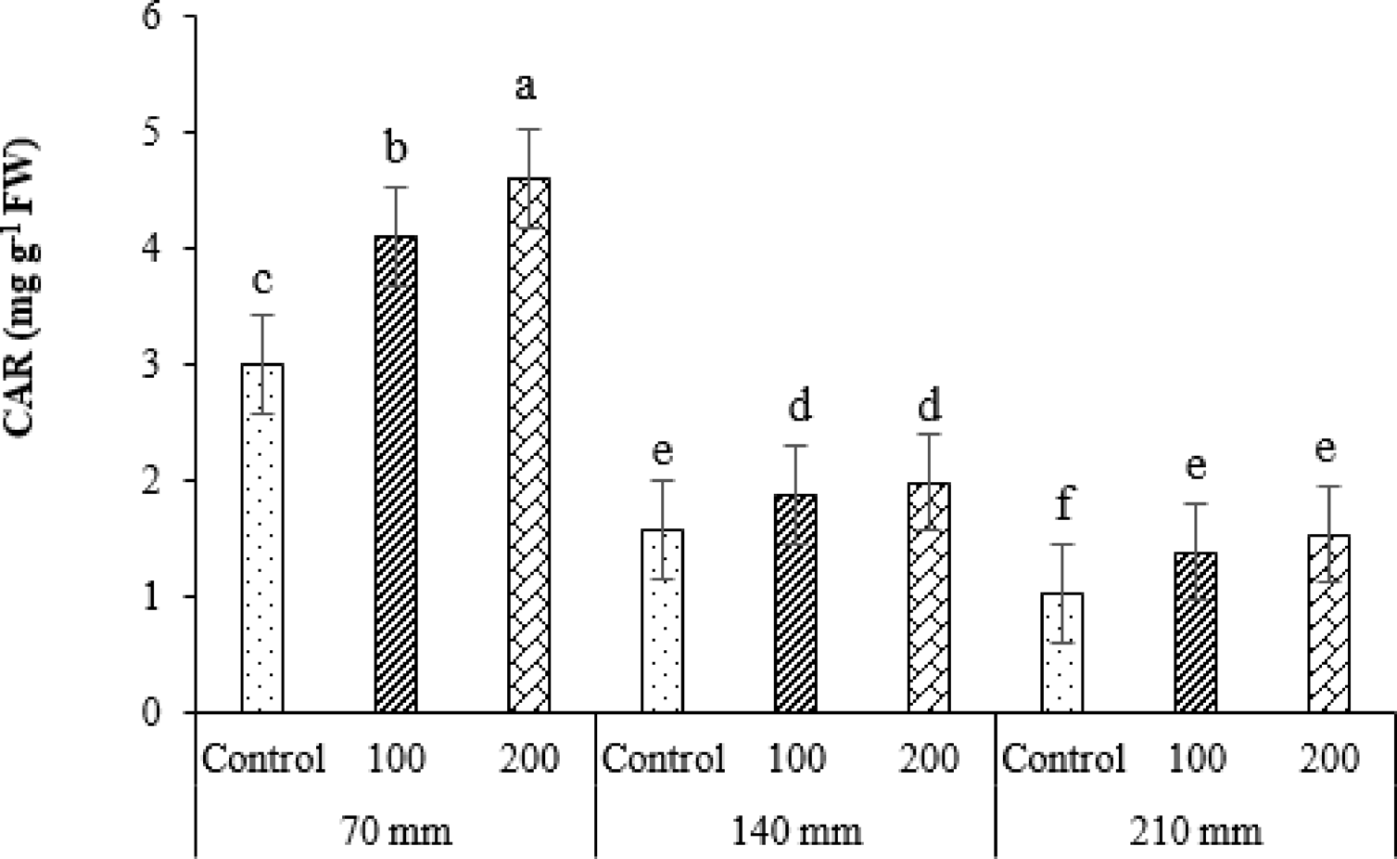
The interaction effect of IR × SA concentrations on the content of CAR (Adapted to the first-year data). Same superscript letters assigned to treatment means in a column indicate non-significant differences from each other at *p* < 0.05. *** Foliar applications included SA at 0 (control), 100, and 200 mg L^− 1^.

Additionally, as data revealed that CAR contents during the first year of the experiment significantly affected by the interaction of IR × SA concentrations, mean comparisons (Fig. 8) indicated that the highest carotenoid content in the first year of the study (60.4 mg g^− 1^ FW) was observed under the 70 mm of evaporation × 200 mg L^− 1^ SA interaction, which was significantly different from other interactions. Conversely, the lowest significant carotenoid content (10.3 mg g^− 1^ FW) was recorded under the interaction of 210 mm of evaporation × no SA (Fig. 8). The combination of 70 mm evaporation and SA appears to synergistically enhance carotenoid accumulation, likely by alleviating drought-induced oxidative stress, stabilizing chloroplast membranes, and promoting photoprotective mechanisms, whereas the absence of these treatments under severe drought stress limits carotenoid synthesis.

**Fig. 9:**
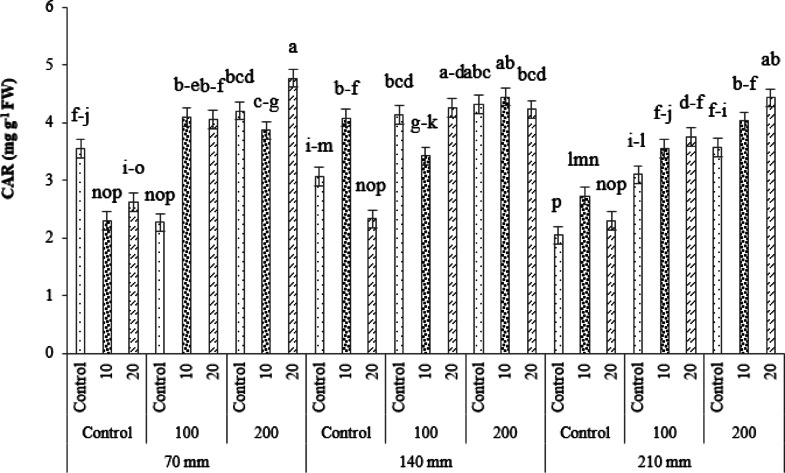
Effects of the three-way interaction of IR × SA concentrations × methanol levels on the content of CAR (Adapted to the second-year data). Same superscript letters assigned to treatment means in a column indicate non-significant differences from each other at *p* < 0.05. *** Foliar applications included SA at 0 (control), 100, and 200 mg L^− 1^ and methanol at 0 (control), 10, and 20% (v/v).

Regarding the significant three-way interaction of IR × SA × methanol on the CAR content (Fig. 9; Second-year data), mean comparisons showed that the highest and lowest carotenoid contents were observed in plants grown under the 70 mm of evaporation × 200 mg L^− 1^ SA × 20% methanol interaction and the interaction of 210 mm of evaporation × non-SA application × non-methanol application, estimated at 4.77 and 2.05 mg g^− 1^ FW, respectively. This pattern suggests that the combination of low water stress, high SA, and methanol enhances carotenoid synthesis and antioxidant defense, whereas severe stress without chemical treatments limits carotenoid accumulation and the plant’s capacity to cope with oxidative stress.

#### Leaf proline content (LPC)

Analysis of variance for the effects of the experimental treatments on selected biochemical traits (Table 5) indicated that IR, SA concentrations, and their interaction had significant impacts on LP (*p < 0.01*) in the first year of the experiment. In the second year of the study, significant differences for LP were observed due to the effects of IR (*p < 0.01*) and IR × SA interaction (*p < 0.05*).

**Table 5:** Analysis of variance (mean square) of the effects of irrigation regime, SA concentrations, and methanol levels on enzymatic and non-enzymatic antioxidants of rapessed.

Source of variation	df	First year data					Second year data				
		LPC	SC	CAT	GPX	APX	LPC	SC	CAT	GPX	APX
Block ^O^	2	0.005ns	2.87ns	0.0006ns	0.001ns	0.03ns	0.52ns	1.46ns	0.00004ns	0.00003ns	0.06ns
Irrigation regimes (IR)	2	8.04**	900.64**	0.104**	0.21**	15.07**	150.8**	22968.13**	0.154**	0.232**	28.38**
Error a (Ea)^●^	4	0.47	1.08	0.0007	0.001	0.07	0.52	23.95	0.002	0.006	0.05
SA concentrations	2	4.33**	461.05**	0.006**	0.016**	0.55**	3.06ns	48.78ns	0.026**	0.339**	22.36**
Methanol levels	2	0.45ns	144.06**	0.003**	0.18**	1.17**	0.11ns	40.76ns	0.01**	0.065**	4.15**
IR × SA	4	2.33**	13.65ns	0.001*	0.001ns	0.07ns	3.65*	648.77**	0.001ns	0.003ns	0.31ns
IR × Methanol	4	0.28ns	1.89ns	0.0004ns	0.0006ns	0.02ns	2.36ns	55.69ns	0.0005ns	0.001ns	0.11ns
SA × Methanol	4	0.05ns	34.39**	0.0002ns	0.0002ns	0.03ns	1.24ns	30.17ns	0.0007ns	0.004ns	0.19ns
IR × SA × Methanol	8	0.06ns	7.99ns	0.0001ns	0.0005ns	0.0008ns	0.27ns	36.58ns	0.0005ns	0.001ns	0.09ns
Error b (Eb)^■^	48	0.17	8.02	0.0003	0.001	0.06	1.14	30.65	0.001	0.007	0.31
CV (%)		12.43	7.29	9.88	8.36	8.04	16.83	9.33	7.53	11.24	12.65

ns, *, and **; non-significant, significant at *p* < 0.05 and significant at *p* < 0.01, respectively.

O Block: experimental replication.

● Ea: experimental error for the main-plot factor (Block × Treatment A).

■ Eb: experimental error for the subplot factor (Block × Treatment A × Treatment B).

Irrigation regimes (IR) represent cumulative evaporation levels of 70, 140, and 210 mm during the growing season. Foliar applications included SA at 0 (control), 100, and 200 mg^− 1^, and methanol at 0 (control), 10, and 20% (v/v).

**Fig. 10:**
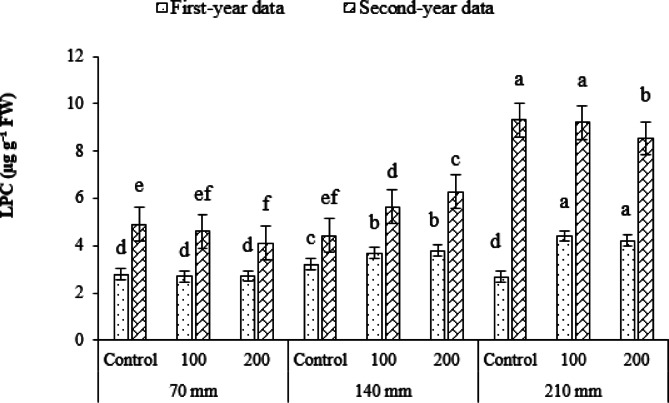
Effects of the two-way interaction of IR × SA concentrations on the content of LP. Same superscript letters assigned to treatment means in a column indicate non-significant differences from each other at *p* < 0.05. *** Foliar applications included SA at 0 (control), 100, and 200 mg L^− 1^.

Concerning the significant interaction of IR × SA concentrations on LPC in rapeseed during the first year of the experiment (Fig. 10), mean comparisons showed that the highest values (4.42 and 4.22 µg g^− 1^ FW), which were not significantly different from each other, were observed under the interactions of 210 mm of evaporation × 100 mg L^− 1^ SA and 210 mm of evaporation × 200 mg L^− 1^ SA, respectively. Conversely, the lowest LPC (2.68 µg g⁻¹ FW) was recorded under the interaction of 210 mm × no SA (Fig. 10; based on the first-year data). Second-year data on LPC revealed that the highest value (9.2 µg g^− 1^ FW) was recorded under the interaction of 210 mm × no SA. However, this value was not significantly different from that observed under 210 mm of evaporation × 100 mg L^− 1^ SA (8.54 µg g^− 1^ FW). The lowest LPC (4.12 µg g^− 1^ FW) was calculated for the interaction of 70 mm of evaporation × 200 mg L^− 1^ SA (Fig. 10; Adapted to the second-year data). Effects of the significant interaction between IR and SA application on LPC indicate that SA mitigates drought-induced lipid peroxidation under high water stress (210 mm), whereas under milder stress or sufficient water, SA reduces LPC, reflecting lower oxidative damage. This demonstrates that the protective role of SA on membrane integrity is modulated by water availability.

#### Soluble carbohydrates (SC)

The analysis of variance for the SC content in rapeseed leaves (Table 5) showed that in the first year, this trait was significantly affected by IR, foliar application of SA, methanol levels, and the two-way interaction of SA concentrations × methanol levels (*p < 0.01*). However, in the second year, the IR and the interaction of IR × SA concentrations had significant effects on the SC content (*p < 0.01*; Table 5).

**Fig. 11:**
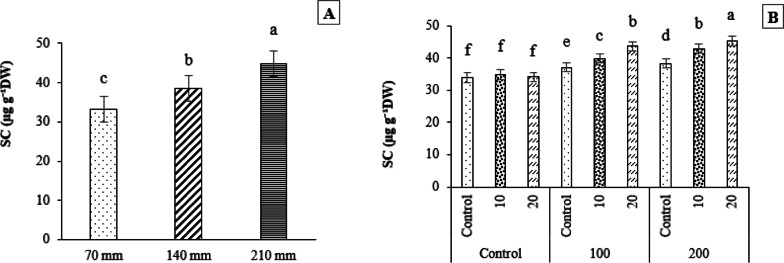
Effects of (**a**) IR and (**b**) the interaction of SA concentrations × Methanol levels on the content of SC (Adapted to the first-year data). Same superscript letters assigned to treatment means in a column indicate non-significant differences from each other at *p* < 0.05. *** Foliar applications included SA at 0 (control), 100, and 200 mg L^− 1^ and methanol at 0 (control), 10, and 20% (v/v).

Mean comparisons of plant responses to IR with respect to SC during the first year of the experiment (Fig. 11a) showed that the highest SC content (44.82 µg g^− 1^ DW) was obtained under the 210-mm evaporation regime, which was significantly higher than those recorded under the 70 and 140 mm evaporation regimes, representing increases of 34.65% and 17.53%, respectively (33.29 and 38.50 µg g^− 1^ DW). Owing to the significant SA × methanol interaction effect on SC (Fig. 11b; first-year data), the maximum SC content (45.31 µg g^− 1^ DW) was observed under the combined application of 200 mg L^− 1^ SA and 20% methanol, which was significantly higher than all other treatment combinations. In contrast, the lowest SC content (33.85 µg g^− 1^ DW) was recorded in the absence of both SA and methanol application; however, this value did not differ significantly from those obtained under no SA × 10% methanol (34.81 µg g^− 1^ DW) and no SA × 20% methanol (34.07 µg g^− 1^ DW). The increased SC accumulation under the combined 200 mg L^− 1^ SA × 20% methanol treatment suggests a synergistic effect of these foliar applications in enhancing osmotic adjustment and maintaining carbon reserves under stress conditions, whereas the relatively low SC levels in untreated plants indicate a limited capacity for stress mitigation and metabolic buffering.

**Fig. 12:**
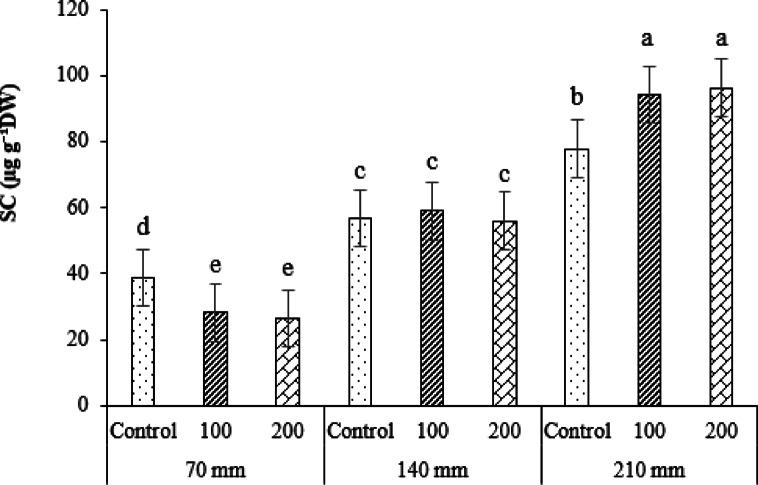
Effects of the interaction of IR × SA concentrations on the content of SC (Adapted to the second-year data). Same superscript letters assigned to treatment means in a column indicate non-significant differences from each other at *p* < 0.05. *** Foliar applications included SA at 0 (control), 100, and 200 mg L^− 1^.

As shown in Table 5, the SC content in the second year of the experiment was significantly affected by the interaction of IR × SA concentrations. Mean comparisons revealed that the highest values (96.25 and 94.20 µg g⁻¹ DW) were recorded under the interactions of 210 mm of evaporation × 200 mg L⁻¹ SA and 210 mm of evaporation × 100 mg L^− 1^ SA, respectively, with no statistically significant difference between them. In contrast, the lowest soluble carbohydrate content (26.49 µg g⁻¹ DW) was recorded under the interaction of 70 mm of evaporation × 200 mg L⁻¹ SA (Fig. 12). Here, the pronounced accumulation of SC under the 210 mm × high concentrations of SA indicates that under elevated irrigation stress, SA may enhance carbon partitioning and osmotic adjustment, helping plants maintain metabolic stability. In contrast, the minimal SC under 70 mm × 200 mg L⁻¹ SA suggests that under less water-limiting conditions, excess SA does not further stimulate carbohydrate accumulation and may even shift metabolism toward growth rather than osmotic protection.

#### Enzymatic activities of CAT, GPX, and APX

##### CAT activity

The related ANOVA data (Table 5) indicated that CAT activity was significantly affected by the experimental treatments. In the first year, catalase activity significantly influenced by IR, SA concentrations, and methanol levels (*p < 0.01*), as well as by the interaction between IR × SA concentrations (*p < 0.05*). In contrast, during the second year, the main effects of the treatments (i.e., IR, SA, and methanol) were significant at *p* < 0.01, whereas no significant differences were observed in CAT activity.

**Fig. 13:**
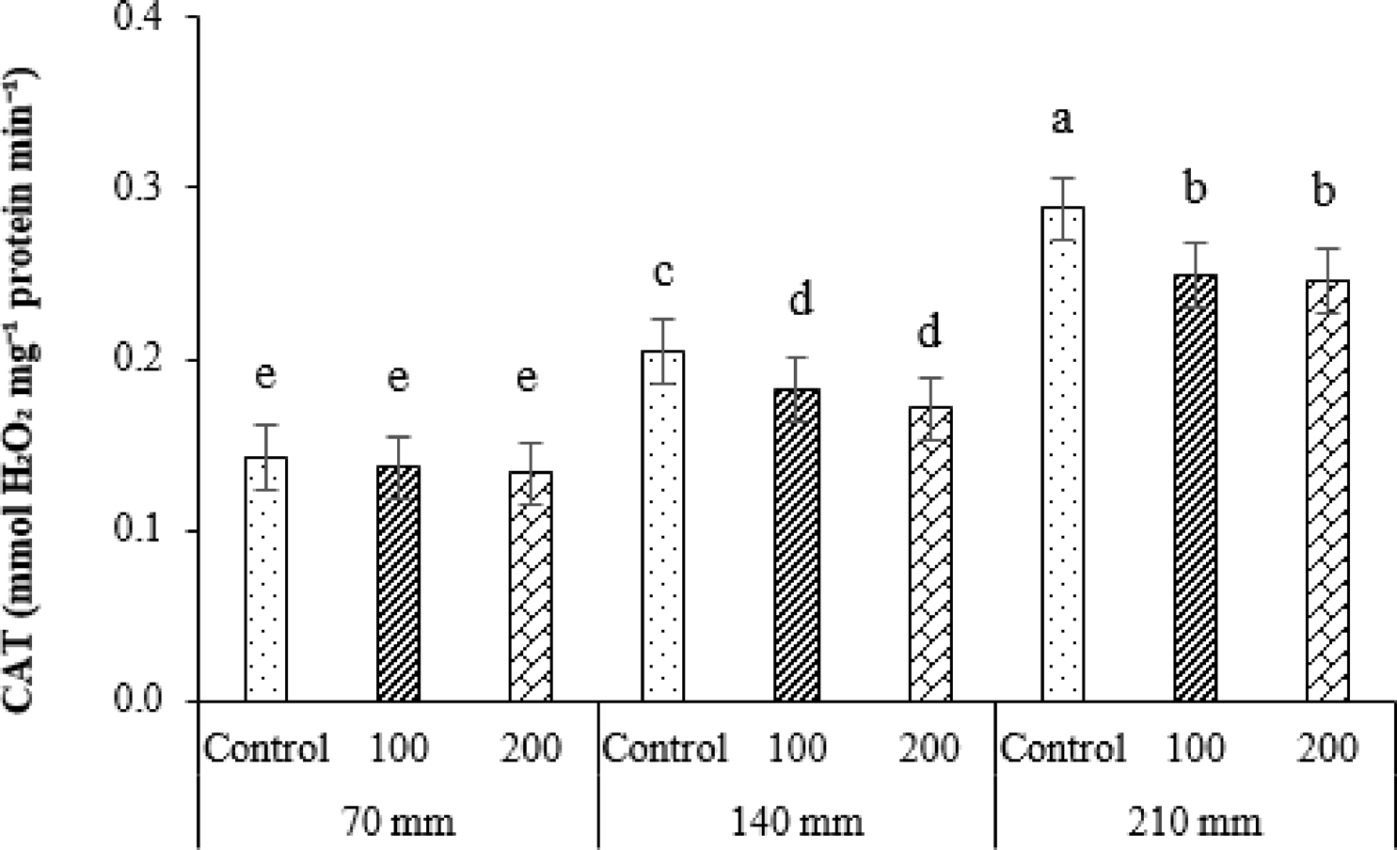
Effects of the interaction of IR × SA concentrations on the enzymatic activity of CAT (Adapted to the first-year data). Same superscript letters assigned to treatment means in a column indicate non-significant differences from each other at *p* < 0.05. *** Foliar applications included SA at 0 (control), 100, and 200 mg L^− 1^.

According to the mean comparisons (Fig. 13; based on the first-year data), the highest CAT activity (0.288 mmol H₂O₂ mg^− 1^ protein min^− 1^) was recorded under the interaction of 210 mm × no SA. Conversely, the lowest CAT activities (0.133, 0.137, and 0.142 mmol H₂O₂ mg^− 1^ protein min^− 1^) were recorded under the interactions of 70 mm × 200 mg L^− 1^ SA, 70 mm × 100 mg L^− 1^ SA, and 70 mm × no SA, respectively. These three combinations did not differ significantly from one another (Fig. 13; first-year data). The pronounced CAT activity under 210 mm × no SA indicates that plants experiencing severe water stress mobilize their enzymatic antioxidant machinery to a maximal extent to scavenge H_2_O_2_. In contrast, lower CAT activities under 70 mm irrigation, regardless of SA application, suggest that under less stressful conditions, SA may enhance non-enzymatic antioxidant pathways or fine-tune ROS signaling, thereby reducing the reliance on CAT. This pattern reflects an intricate balance between stress intensity and hormonal modulation of the antioxidant system in rapeseed.

As shown in the second-year results (see Table 3), drought stress had a significant and direct relationship with increased CAT activity. Mean comparisons of irrigation treatments revealed that the highest and lowest significant CAT activities (0.543 and 0.390 mmol H_2_O_2_ mg^− 1^ protein min^− 1^) occurred under 210 mm and 70 mm of evaporation regimes, respectively. In other words, applying a stress level equivalent to 210 mm of evaporation led to a 39.23% and 8.17% increase in the CAT activity compared to the 70 mm and 140 mm IR. Furthermore, the highest CAT activity under SA foliar treatments (0.509 mmol H_2_O_2_ mg^− 1^ protein min^− 1^) was recorded in the control treatment (no SA). However, CAT activity significantly decreased in response to the 100 and 200 mg L^− 1^ SA concentrations, which could indicate a regulatory effect on enzymatic balance and improved stress adaptation in treated rapeseed plants. Similarly, foliar application of different methanol concentrations revealed that the lowest CAT activity (0.495 mmol H_2_O_2_ mg^− 1^ protein min^− 1^) was observed in the control (no methanol) treatment (see Table 3).

##### GPX activity

According to the ANOVA results (Table 5), all three individual treatments, IR, SA concentrations, and methanol levels, significantly affected GPX activity in both years of the experiment (*p < 0.01*). However, none of the two-way or three-way interactions had a statistically significant impact on GPX activity in either year.

As expected, there was a direct and significant correlation between increasing drought stress and enhanced GPX enzymatic activity. The highest and lowest significant GPX activities in both the first (0.491 and 0.321 U mg⁻¹ protein min⁻¹) and second years (0.491 and 0.321 U mg⁻¹ protein min⁻¹) were observed under the 210 mm and 70 mm of evaporation regimes, respectively. Overall, GPX activity under the 210 mm treatment was significantly higher by 52.96% and 9.60% compared to the 70 mm and 140 mm regimes in the first year, and by 28.77% and 14.36% in the second year, respectively (see Table 3).

Concerning the foliar application of SA on enzymatic activities (see Table 3), data showed that GPX was significantly reduced with increasing SA concentrations across both years. The highest enzymatic activity of GPX was obtained in the first and second years of the study for plants exposed to the control treatment, with average values of 0.449 and 0.856 U mg^− 1^ protein min^− 1^, respectively. In general, compared to the control, SA treatments of 100 and 200 mg L^− 1^ led to significant reductions of 9.25% and 11.69% in the first year and 22.29% and 33.95% in the second year, respectively. Similarly, GPX activity declined in both years following the methanol application. Based on the first-year data, enzymatic activity values for control (no methanol application), 10%, and 20% methanol treatments were 0.448, 0.415, and 0.398 U mg^− 1^ protein min^− 1^, respectively. In addition, the respective values were 0.788, 0.712, and 0.696 U mg^− 1^ protein min^− 1^ in the second year of the experiment.

##### APX activity

APX activity showed significant responses to the main effects of irrigation regime, SA concentration, and methanol level across both years of the study (*p < 0.01*). However, no statistically significant interactions were detected among these factors (Table 5).

In the first year, APX activity was highest under the 210 mm evaporation regime (3.88 U mg^− 1^ protein min^− 1^), representing significant increases of 50.89% and 2.74% compared to 70 mm and 140 mm IR, respectively (Table 3). Regarding SA treatments, APX activity decreased with increasing SA concentration, with values of 3.23, 3.02, and 2.96 U mg^− 1^ protein min^− 1^ under control, 100, and 200 mg L^− 1^ SA, respectively, corresponding to reductions of 6.50% and 8.36% relative to the control. Similarly, methanol application reduced APX activity, with the highest activity observed under the control (3.30 U mg^− 1^ protein min^− 1^) and lower activities under 10% methanol (3.02 U mg^− 1^ protein min^− 1^) and 20% methanol (2.89 U mg^− 1^ protein min^− 1^), representing decreases of 9.27% and 14.19% compared to the control (see Table 3).

According to the second-year data, APX activity was significantly affected by IR, SA, and methanol treatments. The highest APX activity was observed under the IR regime of 210 mm (5.35 U mg^− 1^ protein min^− 1^), and plants subjected to 70 mm and 140 mm IR exhibited markedly lower activities (3.36 and 4.44 U mg^− 1^ protein min^− 1^, respectively). Also, increasing SA concentration significantly reduced APX activity, with the control showing the highest value (5.38 U mg^− 1^ protein min^− 1^). Similarly, methanol application negatively affected APX activity, as the control treatment exhibited the highest enzymatic activity compared with both 10% and 20% methanol treatments (see Table 3).

### Yield and yield-related characteristics

#### Silique per plant (SPP) and grain per silique (GPS)

The analysis of variance for the main effects and two- and three-way interactions on SPP (Table 6) indicated that the SPP trait was significantly influenced by IR, SA concentrations, and methanol levels at *p* < 0.01 in both years of the study. Furthermore, according to the analysis of variance, IR and foliar application of different concentrations of SA had significant effects on GPS in both experimental years (*p < 0.01*).

**Table 6:** Analysis of variance (mean square) of the effects of irrigation regime, SA concentrations, and methanol levels on yield-related traits of rapeseed.

Source of variation	df	SPP		GPS		TGW		GY		OP		OY	
		First year data	Second year data	First year data	Second year data	First year data	Second year data	First year data	Second year data	First year data	Second year data	First year data	Second year data
Block ^O^	2	11.7ns	30.74ns	33.05ns	0.92ns	0.003ns	0.04ns	133549.9ns	5162.96ns	62.26ns	6.15ns	52987.18ns	6694.13ns
Irrigation regimes (IR)	2	4585.09**	6551.04**	711.12**	344.54**	3.23**	8.83**	71133967.1**	24159338.59**	686.6**	1000.16**	19931759.21**	4742767.31**
Error a (Ea)^●^	4	83.24	6.49	25.43	6.52	0.04	0.01	160,233	27905.54	1.66	24.32	35095.31	4757.96
SA concentrations	2	500.89**	703.52**	106.55**	1530.77**	0.15ns	0.38**	6444543.2**	3341697.18**	190.63**	158.38**	2380699.43**	644892.86**
Methanol levels	2	523.72**	751.22**	38.17ns	24.01ns	0.11ns	0.23*	5450597.9**	2028268.81**	130.27**	95.6**	1869437.02**	428255.07**
IR × SA	4	24.55ns	22.63ns	11.37ns	12.25ns	0.02ns	0.02ns	211459.9ns	353224.09**	26.15ns	14.38ns	203030.53**	108829.22**
IR × Methanol	4	9.28ns	14.35ns	0.69ns	5.25ns	0.006ns	0.02ns	224834.7ns	206749.84**	7.51ns	2.89ns	178178.62*	81932.23**
SA × Methanol	4	12.09ns	20.52ns	2.65ns	2.79ns	0.01ns	0.04ns	70546ns	91293.79ns	2.73ns	2.96ns	34658.56ns	19242.41ns
IR × SA × Methanol	8	12.21ns	11.1ns	2.34ns	14.05ns	0.02ns	0.02ns	71509.9ns	53850.64ns	2.3ns	1.88ns	16656.93ns	11118.84ns
Error b (Eb)^■^	48	37.17	26.49	19.73	12.54	0.09	0.06	270308.6	53818.46	22.86	17.17	50905.2	10875.11
CV (%)		11.73	14.39	16.46	17.85	8.61	9.89	15.96	18.17	11.41	12.08	15.78	21.48

ns, *, and **; non-significant, significant at *p* < 0.05 and significant at *p* < 0.01, respectively.

O Block: experimental replication.

● Ea: experimental error for the main-plot factor (Block × Treatment A).

■ Eb: experimental error for the subplot factor (Block × Treatment A × Treatment B).

Irrigation regimes (IR) represent cumulative evaporation levels of 70, 140, and 210 mm during the growing season. Foliar applications included SA at 0 (control), 100, and 200 mg^− 1^, and methanol at 0 (control), 10, and 20% (v/v).

Mean comparisons of the effects of IR, SA concentrations, and methanol levels on SPP across both years (see Table 3) illustrated that drought stress negatively affected SPP in both years. In the first year, plants under the 70 mm evaporation treatment exhibited the highest SPP (with an average of 66.16 silique numbers), which was significantly greater than those under 140 mm and 210 mm evaporation (49.23 and 40.54 silique numbers, respectively). In percentage terms, the 70 mm irrigation treatment led to 34.40% and 63.21% increases in SPP compared to the 140 mm and 210 mm regimes, respectively (Fig. 17a; first-year data). Similarly, in the second year, SPP significantly decreased under the 140 mm and 210 mm IR (mean values of 34.47 and 20.85 numbers, respectively) in comparison to the 70 mm treatment by a mean of 51.93 siliques (See Table 3; Adapted to the second year data).

The significant effect of SA concentration on yield components was observed in both years. In the first year, 200 mg L^− 1^ SA produced the highest SPP (56.58 siliques), significantly exceeding the control (48.04) and 100 mg L^− 1^ SA (51.31), corresponding to increases of 17.77% and 10.27%, respectively, while in the second year, the same treatment increased SPP to 41.26 siliques, representing increases of 32.30% and 18.60% relative to the control (31.19) and 100 mg L^− 1^ SA (34.79). Methanol application also affected SPP, with the highest and lowest values in the first year recorded under 20% methanol (56.77) and control (48.12), respectively, whereas 10% methanol (51.04) did not differ significantly from the control; 20% methanol increased SPP by 17.99% and 11.23% relative to the control and 10% methanol. Similarly, in the second year, both 10% and 20% methanol significantly enhanced SPP to 35.84 and 40.97 siliques plant⁻¹ compared to 30.43 in the control, corresponding to increases of 17.79% and 34.65%, respectively (Table 3).

**Fig. 14:**
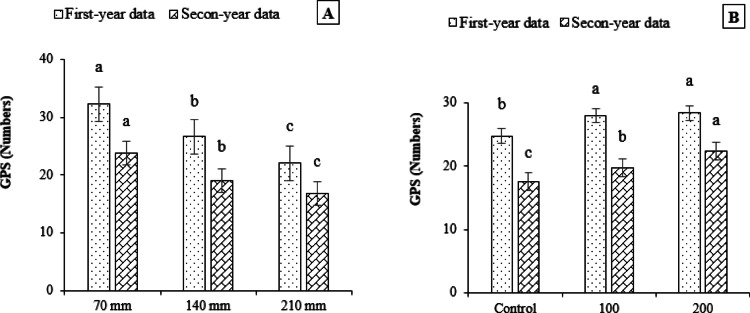
Effects of (**a**) IR and (**b**) SA concentrations on the GPS trait. Same superscript letters assigned to treatment means in a column indicate non-significant differences from each other at *p* < 0.05. *** Foliar applications included SA at 0 (control), 100, and 200 mg L^− 1^.

The mean comparisons for the effect of IR on GPS showed that in the first year, the highest GPS (32.3 grains) was recorded under the 70 mm evaporation treatment, representing significant increases of 21.34% and 46.48% compared to the 140 mm and 210 mm treatments (26.62 and 22.05 grains, respectively; Fig. 14a). In the second year, the 70 mm evaporation regime again produced the highest GPS (23.76 grains), while the 210 mm treatment resulted in the lowest value (16.75 grains), corresponding to increases of 24.87% and 41.79% relative to the 140 mm and 210 mm treatments (Fig. 14a). Foliar application of SA also significantly affected GPS in both years. In the first year, the highest and lowest GPS values (28.33 and 24.71 grains) were obtained under 200 mg L^− 1^ SA and the control, respectively, indicating increases of 14.68% and 1.46% relative to the control and 100 mg L^− 1^ SA. Similarly, in the second year, 200 mg L^− 1^ SA produced the highest GPS (23.76 grains), while the control resulted in the lowest value (16.75 grains; Fig. 14b).

#### Thousand-grain weight (TGW)

The ANOVA results related to the effects of experimental treatments on TGW (Table 6) indicated that IR had significant effects on this trait in the first year of the experiment (*p < 0.01*). However, the results obtained from the second-year data were somewhat different, showing that TGW was significantly influenced by IR and SA concentrations (*p < 0.01*), as well as methanol levels (*p < 0.05*). Nonetheless, the effects of two-way and three-way interactions among treatments on TGW were negligible and non-significant in both years.

**Fig. 15:**
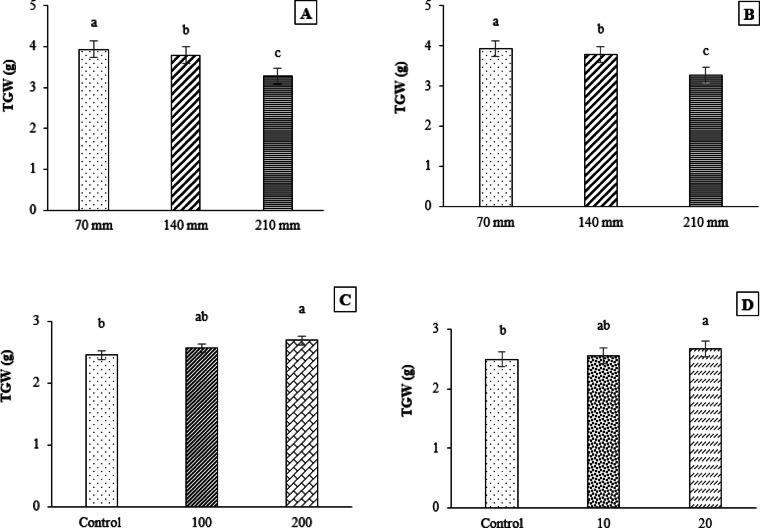
Changes of TGW under (**a**) IR (first-year data), and (**b**) IR, (**c**) SA concentrations, and (**d**) methanol levels (second-year data). Same superscript letters assigned to treatment means in a column indicate non-significant differences from each other at *p* < 0.05. *** Foliar applications included SA at 0 (control), 100, and 200 mg L^− 1^ and methanol at 0 (control), 10, and 20% (v/v).

Based on the mean comparison squares (Fig. 15a; first year data), the highest TGW value (3.93 g) was observed under the 70 mm evaporation irrigation regime. Data analysis suggests that TGW values for plants grown under the 70 mm evaporation regime increased by 20.05% and 3.08% compared to those treated with 140 mm and 210 mm evaporation regimes, respectively.

Mean comparison results of the simple effects of treatments on TGW in the second-year data (Fig. 15b–d) revealed that the highest TGW value (3.1 g) was obtained under the 70 mm evaporation irrigation regime, which showed a significant increase compared to other irrigation treatments. The lowest TGW (1.96 g) was recorded for plants under the 210 mm evaporation regime (Fig. 15b). Regarding the effects of different SA concentrations on TGW in the second-year data, the highest and lowest TGW values (2.69 g and 2.46 g) were recorded for the 200 mg L^− 1^ SA treatment and the control, respectively. In other words, TGW increased by 9.7% and 5.04% under 200 mg L^− 1^ SA compared to the control and 100 mg L^− 1^ SA treatments, respectively (Fig. 15c). Furthermore, mean comparisons related to foliar application of different methanol levels (Fig. 15d) indicated that the highest TGW value (2.67 g) was obtained from the 20% methanol treatment, with no significant difference compared to the 10% methanol treatment (2.55 g). The lowest TGW value (2.49 g) was observed in the control group (no methanol application). Overall, it can be concluded that the application of 20% methanol increased TGW by 7.23% and 4.51% compared to the control and 10% methanol treatments, respectively.

#### Grain yield (GY)

The ANOVA results for the effects of experimental treatments on GY are presented in Table 6, indicating that the simple treatments (IR, SA concentrations, and various methanol levels) had significant effects on GY in the first year of the experiment at *p* < 0.01. Moreover, the second-year data revealed significant effects of IR, SA concentrations, methanol levels, as well as the interactions of IR × SA concentration and IR × methanol levels on GY (*p < 0.01*).

**Fig. 16:**
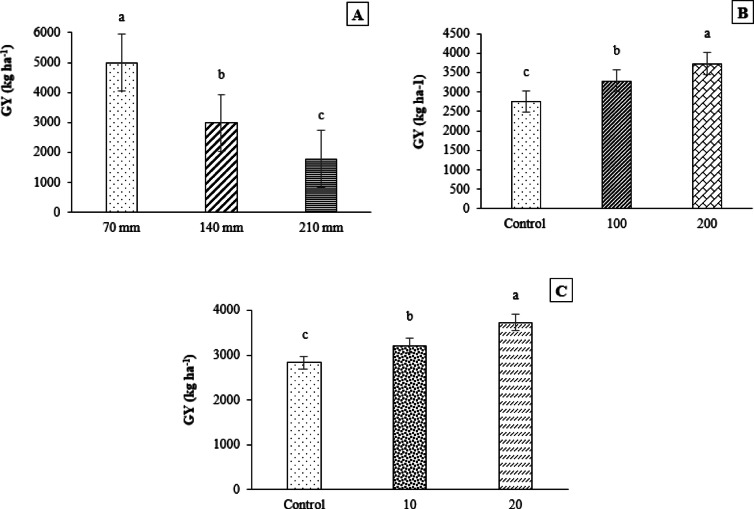
Changes of GY under (**a**) IR, (**b**) SA concentrations, and (**c**) methanol levels (Adapted to the first-year data). Same superscript letters assigned to treatment means in a column indicate non-significant differences from each other at *p* < 0.05. *** Foliar applications included SA at 0 (control), 100, and 200 mg L^− 1^ and methanol at 0 (control), 10, and 20% (v/v).

Mean comparisons of the simple treatments in the first year (Fig. 16a–c) showed that the highest GY (1,500.1 kg ha⁻¹) was obtained under the 70 mm evaporation irrigation regime, which was significantly higher than the GY recorded in other treatments. The lowest GY (1,790 kg ha^−^¹) was observed under the 210 mm evaporation regime (Fig. 16a). Considering the significant effects of different concentrations of SA on GY (Fig. 16b), the highest value was estimated for the 200 mg L^− 1^ SA concentration (with an average of 3731.5 kg ha^− 1^), showing a significant increase compared to the control (2,755.7 kg ha⁻¹) and the 100 mg L^− 1^ SA treatment (3,286.8 kg ha⁻¹). Furthermore, foliar application of different methanol levels significantly affected GY, with a direct and significant positive correlation between methanol concentration and rapeseed yield. The highest and lowest GY values under methanol treatments in the first year were 3,727.2 and 2,831.7 kg ha⁻¹, corresponding to 20% methanol and control treatments, respectively (Fig. 16c).

**Fig. 17:**
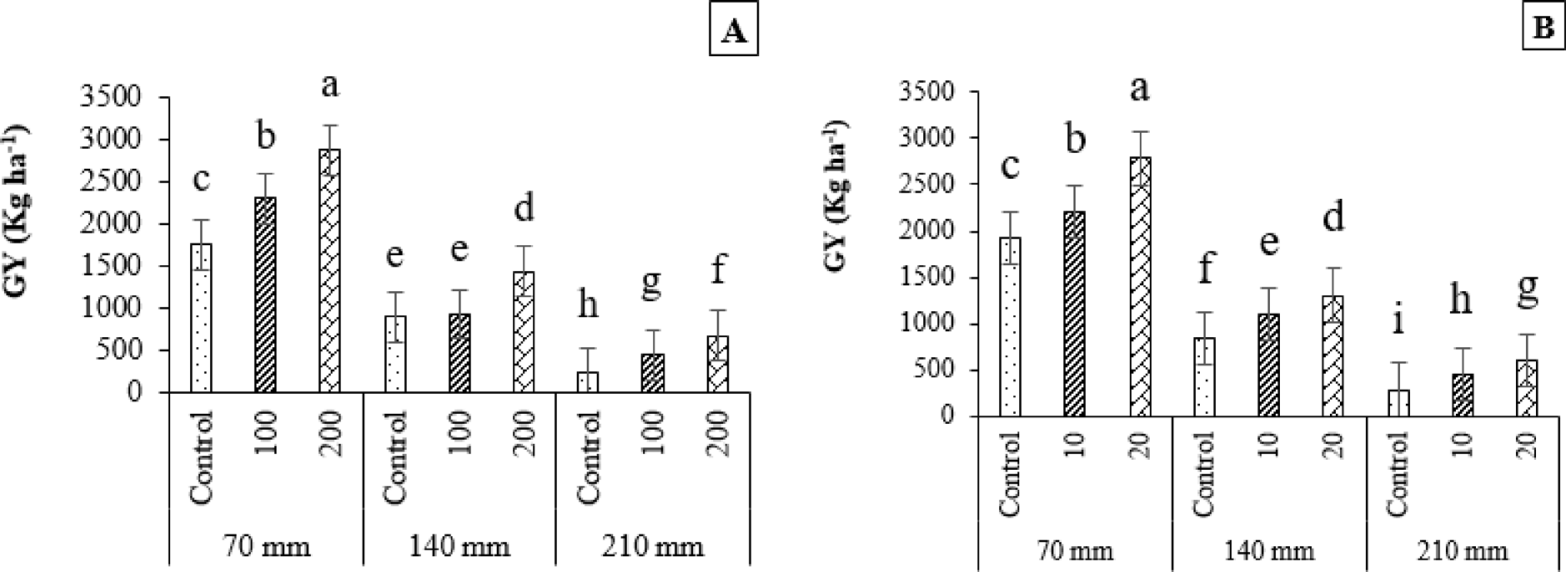
Changes of GY under the interactions of (**a**) IR × SA concentrations and (**b**) IR × methanol levels (Adapted to the first-year data). Same superscript letters assigned to treatment means in a column indicate non-significant differences from each other at *p* < 0.05. *** Foliar applications included SA at 0 (control), 100, and 200 mg L^− 1^ and methanol at 0 (control), 10, and 20% (v/v).

Based on the second-year data, the GY trait was affected not only by simple treatments (Table 6) but also by the interactions of IR × SA concentrations and IR × methanol levels. According to the obtained data, drought stress (i.e., irrigation at higher evaporation levels) significantly decreased rapeseed GY. However, SA and methanol, especially at higher concentrations and levels, partially mitigated the negative effects of drought stress in the present study. Overall, the highest GY in the second-year data was estimated at 2868.45 kg ha⁻¹, recorded under the interaction of the irrigation regime and 200 mg L^− 1^ SA treatment. Conversely, the lowest GY values (2227.2, 436.38, and 667.5 kg ha⁻¹), although significantly different from each other, were recorded under the interactions of 210 mm evaporation × no SA foliar spray, 210 mm evaporation × 100 mg L^− 1^ SA foliar spray, and 210 mm evaporation × 100 mg L^− 1^ SA foliar spray, respectively (Fig. 17A). The results related to the interaction between irrigation regime and foliar application of different methanol levels (Fig. 17B) also indicated that the highest and lowest GY were obtained under the interactions of 70 mm evaporation × 20% methanol (2,656.43 kg ha⁻¹) and 210 mm evaporation × no methanol spray (157.08 kg ha⁻¹), respectively.

#### Oil percentage (OP)

The ANOVA results concerning the effects of experimental treatments on oil-related traits of rapeseed (Table 6) also indicated that oil percentage was significantly affected by all three simple treatments, IR, SA concentrations, and methanol levels, in both years of the experiment at *p* < 0.01. However, none of the examined interactions resulted in significant changes in this trait.

**Fig. 18:**
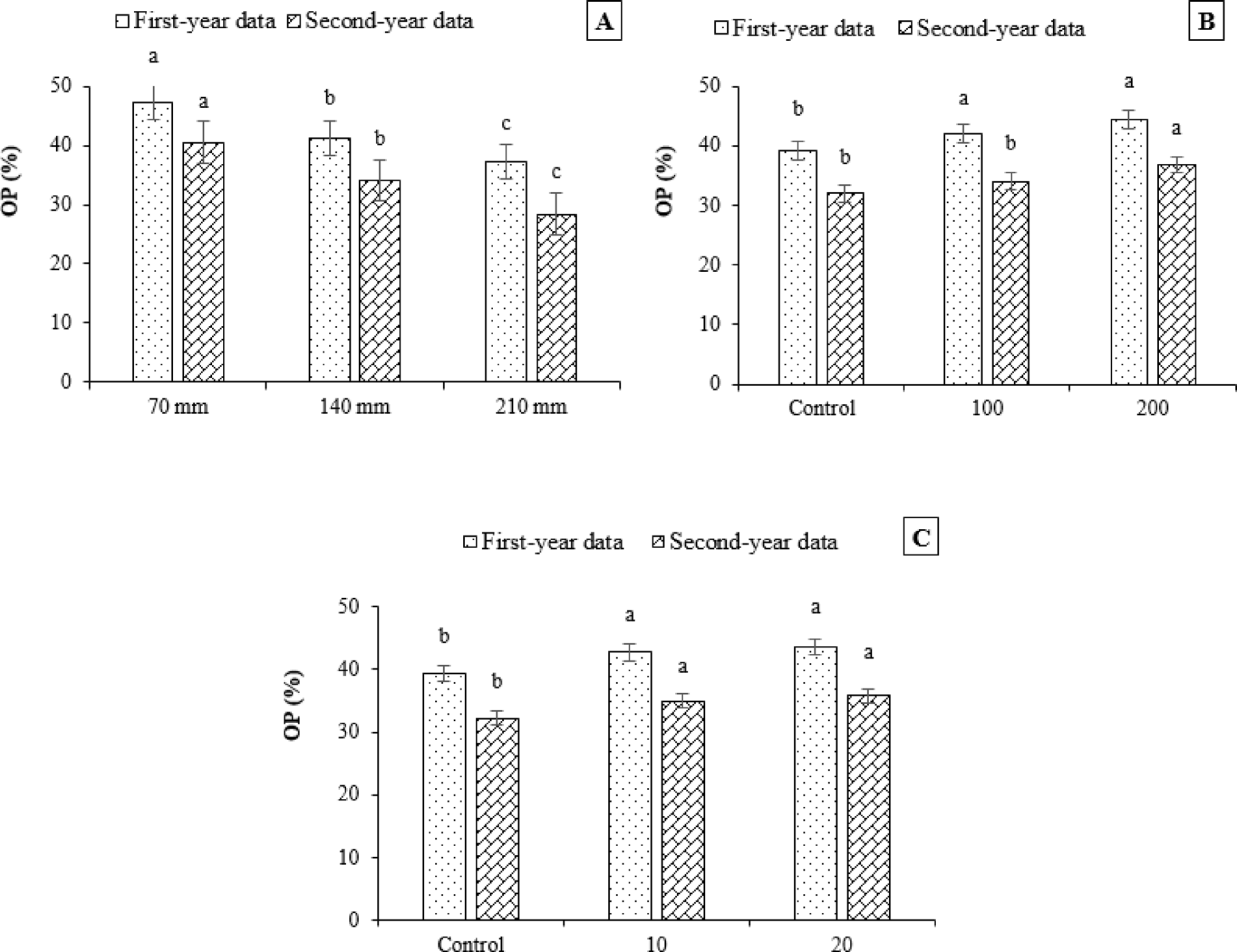
Effects of (**a**) IR, (**b**) SA concentrations, and (**c**) methanol levels on the OP trait. Same superscript letters assigned to treatment means in a column indicate non-significant differences from each other at *p* < 0.05. *** Foliar applications included SA at 0 (control), 100, and 200 mg L^− 1^ and methanol at 0 (control), 10, and 20% (v/v).

Based on the mean comparisons of the first-year data, it was observed that plants treated with the 70 mm evaporation irrigation regime (mean 47.28%) exhibited the highest oil percentage in rapeseed seeds, showing significant increases compared to plants subjected to drought stress under 140 and 210 mm evaporation regimes (with mean values of 41.2% and 37.27%, respectively). According to these data, the increase in oil percentage in plants grown under the 70 mm irrigation regime was estimated at 14.76% and 26.86% relative to the 140 mm and 210 mm IR, respectively (Fig. 18A; first-year data). Additionally, the effects of different SA concentrations revealed that the highest and lowest oil percentages were recorded for plants foliar-sprayed with 200 mg L^− 1^ SA and the control treatment, averaging 44.48% and 39.17%, respectively (Fig. 18B; first-year data). Furthermore, under methanol application, the highest OP was obtained for 20% methanol (43.59%), showing no significant difference compared to the 10% methanol treatment (42.74%). The lowest oil percentage was recorded in the control plants without methanol application, estimated at 39.43% (Fig. 18C; first-year data).

Mean comparisons of the effects of experimental treatments on OP in the second-year data (see Fig. 18a–c; second-year data) showed that application of the 70 mm evaporation irrigation regime (mean 40.53%) significantly increased oil percentage compared to drought stress regimes at 140 and 210 mm evaporation (with mean values of 34.05% and 28.37%), representing increases of 19.03% and 42.86%, respectively (Fig. 18a; second-year data). Moreover, to alleviate drought stress effects, similar to the first year, various proportions of SA and methanol were foliar-applied, and the results showed that spraying with 200 mg L^− 1^ SA significantly increased oil percentage by 15.08% and 8.1% compared to control and 100 mg L^− 1^ SA treatments, respectively (Fig. 18a; second-year data). In addition, the obtained data concluded that 20% methanol (mean 35.82%) significantly increased OP compared to the control treatment (mean 32.21%). However, the increase in oil percentage under 20% methanol compared to 10% methanol (mean 34.93%) was not statistically significant (Fig. 18c; second-year data).

#### Oil yield (OY)

According to the analysis of variance results presented in Table 6, the OY data in the first year of the study was significantly affected by irrigation regime, SA concentrations, methanol levels, the interaction of IR × SA concentrations (at *p* < 0.01), and the interaction between irrigation regime × methanol levels (at *p* < 0.05). In the second year, OY was significantly influenced by irrigation regime, various SA concentrations, different methanol levels, the interaction of irrigation regime × SA concentrations, and the interaction of irrigation regime × methanol levels at *p* < 0.01, leading to significant variations in OY.

**Fig. 19:**
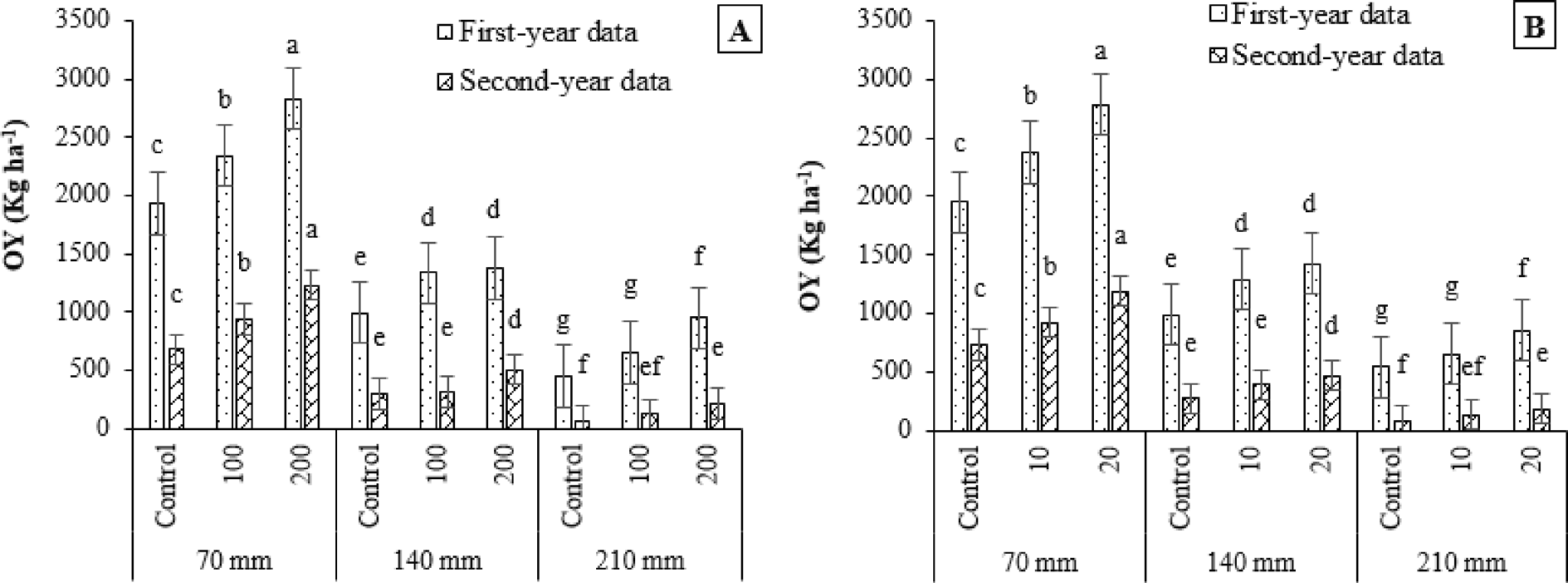
Changes of OY under the interactions of (**a**) IR × SA concentrations and (**b**) IR × methanol levels. Same superscript letters assigned to treatment means in a column indicate non-significant differences from each other at *p* < 0.05. *** Foliar applications included SA at 0 (control), 100, and 200 mg L^− 1^ and methanol at 0 (control), 10, and 20% (v/v).

Based on the first-year data (Fig. 19a), mean comparisons of the IR × SA interaction showed that the highest OY (2831.92 kg ha^− 1^) was obtained under 70 mm evaporation × 200 mg L^− 1^ SA, which was significantly higher than all other treatment combinations. In contrast, the lowest OY values were recorded under 210 mm evaporation combined with no SA (449.44 kg ha⁻¹) and 100 mg L^− 1^ SA (655.34 kg ha^− 1^). A similar response pattern was observed in the second year, where the highest OY (1230.18 kg ha^− 1^) again resulted from the interaction of 70 mm evaporation × 200 mg L^− 1^ SA, whereas the lowest yields were associated with 210 mm evaporation combined with no SA, 100 mg L^− 1^ SA, and 200 mg L^− 1^ SA, producing 57.69, 124.26, and 214.94 kg ha^− 1^, respectively (Fig. 19a).

Mean comparisons of OY under the IR × methanol interaction (Fig. 19b) showed that, in both years, the highest OY was obtained under 70 mm evaporation × 20% methanol, with a mean of 2782.24 kg ha-1, significantly higher than all other treatment combinations. In the first year, the lowest OY values (544.11, 855.79, and 855.79 kg ha^− 1^) were recorded under 210 mm evaporation combined with the control, 10% methanol, and 20% methanol treatments, respectively. Similarly, in the second year of the experiment, the highest and lowest OY values (1193.22 and 81.43 kg ha^− 1^) were observed under 70 mm evaporation × 20% methanol and 210 mm evaporation × no methanol application, respectively (Fig. 19b).

### Correlation of traits with GY and OY

The correlations between the evaluated traits were analyzed separately for each year of the present study, and the results are presented in (Tables 7 and 8). Based on the obtained data (based on the first-year data), GY exhibited significant positive correlations with TGW, RWC, and SPP (respectively). In addition, OY indicated a high positive correlation with GY and TGW and a negative correlation with GPS (Table 7; based on the first-year data). According to the second-year data, the GY trait also showed the highest positive correlation with TGW, RWC, and Chl content. The OY trait also had the highest positive correlation with GY, TGW, RWC, and Chl content, respectively, and a negative correlation with GPS (Table 8).

**Table 7:** Correlation of the studied traits with GY and OY. (Adapted from the first-year data of the study).

Investigated traits	PHt (cm)	Chl T (mg g⁻¹ FW)	RWC (%)	SPP (Numbers)	GPS (Numbers)	SL (cm)	TGW (g)	GY (kg ha^− 1^)	OY (kg ha^− 1^)
PHt (cm)	1.00								
Chl T (mg g⁻¹ FW)	0.88723 *< 0.001*	1.00							
RWC (%)	0.93961 *< 0.001*	0.64285 *< 0.001*	1.00						
SPP (Numbers)	0.67586 *< 0.001*	0.69441 *< 0.001*	0.67025 *< 0.001*	1.00					
GPS (Numbers)	0.59085 *< 0.001*	0.67143 *< 0.001*	0.79526 *< 0.001*	0.55102 *< 0.001*	1.00				
SL (cm)	0.72714 *< 0.001*	0.66079 *< 0.001*	0.86649 *< 0.001*	0.65601 *< 0.001*	0.66452 *< 0.001*	1.00			
TGW (g)	0.57958 *< 0.001*	0.61209 *< 0.001*	0.76253 *< 0.001*	0.61408 *< 0.001*	0.50447 *< 0.001*	0.51806 *< 0.001*	1.00		
GY (kg ha^− 1^)	0.77034 *< 0.001*	0.78462 *< 0.001*	0.86691 *< 0.001*	0.82586 *< 0.001*	0.69709 *< 0.001*	0.64308 *< 0.001*	0.97053 *< 0.001*	1.00	
OY (kg ha^− 1^)	0.60833 *< 0.001*	0.72937 *< 0.001*	0.86239 *< 0.001*	0.81408 *< 0.001*	-0.72039 *< 0.001*	0.51746 *< 0.001*	0.88607 *< 0.001*	0.93085 *< 0.001*	1.00

**Table 8:** Correlation of the studied traits with GY and OY. (Adapted from the second-year data of the study).

Investigated traits	PHt (cm)	Chl T (mg g⁻¹ FW)	RWC (%)	SPP (Numbers)	GPS (Numbers)	SL (cm)	TGW (g)	GY (kg ha^− 1^)	GY (kg ha^− 1^)
PHt (cm)	1.00								
Chl T (mg g⁻¹ FW)	0.71604 *< 0.001*	1.00							
RWC (%)	0.65563 *< 0.001*	0.54071 *< 0.001*	1.00						
SPP (Numbers)	0.68636 *< 0.001*	0.70238 *< 0.001*	0.58305 *< 0.001*	1.00					
GPS (Numbers)	0.57007 *< 0.001*	0.48593 *< 0.001*	0.67503 *< 0.001*	0.57259 *< 0.001*	1.00				
SL (cm)	0.72406 *< 0.001*	0.60127 *< 0.001*	0.76097 *< 0.001*	0.66601 *< 0.001*	0.64089 *< 0.001*	1.00			
TGW (g)	0.60004 *< 0.001*	0.50893 *< 0.001*	0.63108 *< 0.001*	0.62453 *< 0.001*	0.53108 *< 0.001*	0.66029 *< 0.001*	1.00		
GY (kg ha^− 1^)	0.73196 *< 0.001*	0.83609 *< 0.001*	0.86338 *< 0.001*	0.79817 *< 0.001*	0.57639 *< 0.001*	0.64509 *< 0.001*	0.91512 *< 0.001*	1.00	
OY (kg ha^− 1^)	0.64582 *< 0.001*	0.83102 *< 0.001*	0.83596 *< 0.001*	0.77419 *< 0.001*	-0.64152 *< 0.001*	0.58648 *< 0.001*	0.87526 *< 0.001*	0.92875 *< 0.001*	1.00

## Discussion

As previously stated, drought stress is among the most critical abiotic constraints limiting plant growth, development, metabolism, and yield, particularly in arid and semi-arid regions^39,62^. Plants respond to drought stress through a complex network of morphological, physiological, biochemical, and metabolic adjustments aimed at maintaining homeostasis and sustaining growth^63–67^. Under drought conditions, impaired water availability causes stomatal closure, limiting CO_2_ diffusion and reducing photosynthetic carbon assimilation. Reduced CO_2_ availability decreases NADPH consumption and disrupts the balance of the photosynthetic electron transport chain, which leads to an over-reduction of electron carriers and the enhanced production of ROS, such as hydrogen peroxide and superoxide. These ROS can cause oxidative damage to cellular components unless efficiently scavenged by antioxidant systems^68,69^. In the present study, drought stress significantly reduced rapeseed plant height, leaf area, and key physiological traits, including chlorophyll content, proline accumulation, and soluble carbohydrate levels, particularly under the 210 mm evaporation regime^25,70,71^. Osakabe et al.^72^ also emphasized that all stages of plant growth and development exhibit varying degrees of sensitivity to drought stress, depending on the species. Our findings corroborate previous reports indicating that rapeseed is highly susceptible to drought conditions throughout its developmental cycle. These reductions reflect both structural limitations due to reduced turgor and metabolic adjustments as plants prioritize survival over growth; for example, drought-induced ROS accumulation disrupts membrane integrity and can degrade photosynthetic pigments, further limiting carbon fixation^73,74^.

Foliar application of SA mitigated drought-induced damage by enhancing chlorophyll metabolism, photosynthetic efficiency, water status, osmotic adjustment, and antioxidant defense, while also increasing glyoxalase system activity and modulating the ascorbate-glutathione redox cycle, thereby strengthening plant tolerance to drought stress^75–77^. SA, as a signaling molecule, activates enzymatic antioxidants and non-enzymatic systems, modulates stomatal closure, stabilizes chloroplast structure, and promotes the accumulation of osmolytes such as proline and soluble sugars^35,78,79^. Some molecular studies indicated that exogenous SA regulated stress‑responsive genes and stimulated antioxidant enzyme activities, including CAT, SOD, and APX, leading to improved ROS scavenging and the production of cellular components under drought conditions. Specifically, transcriptomic analyses in *Hibiscus cannabinus* (kenaf) have demonstrated that SA application under drought upregulates genes involved in antioxidant pathways alongside increased CAT, SOD, and POD activities^80^. Moreover, SA‑mediated enhancement of redox-related gene expression and antioxidant capacity has been linked to improved ROS detoxification in drought‑affected plants^81^. These responses collectively improve water retention, maintain photosynthetic efficiency, and sustain growth under stress. In the present study, 200 mg L^− 1^ SA significantly increased plant height by 7.81% and 14.53% in the first and second years, respectively, and enhanced leaf relative water content, corroborating previous observations^82–85^. Similar improvements in water status under SA treatment have been linked to enhanced stomatal regulation and osmotic adjustment in other species, supporting the role of SA in maintaining physiological function under drought, e.g., in Toona ciliata where SA application modulated the osmotic system and gas exchange under drought, and in Pisum sativum where SA enhanced stomatal conductance and antioxidative responses^86,87^. While SA has numerous positive effects on plant growth, excessive concentrations can inhibit development, highlighting the need for optimized application strategies^35^. Similarly, foliar application of methanol (10% and 20% v/v) significantly improved morphological and physiological traits in drought-stressed rapeseed. Methanol can serve as an exogenous carbon source, partially compensating for CO_2_ limitation under drought and supporting photosynthesis^88–90^. Methanol may also reduce photorespiration via glycolate oxidase inhibition and rapid oxidation to CO_2_, enhancing Rubisco carboxylation efficiency and prolonging leaf photosynthetic activity^46,91–93^. Additionally, methanol may stimulate methylotrophic leaf bacteria, promoting growth via phytohormone production, and rerouting metabolic flux toward amino acid biosynthesis, similar to observations in microalgae^94^. At the molecular level, SA and methanol may functionally converge on redox homeostasis and signaling pathways, modulating stress-responsive targets to limit oxidative damage^95^. Genetic evidence supports that specific loci underlie stress tolerance traits, linking physiological responses such as plant height, chlorophyll content, and yield components to drought resilience^96^. This integrated physiological response highlights that foliar treatments influence both primary metabolism and stress signaling networks, enhancing overall drought adaptation.

Although methanol application at low concentrations (such as the 10% and 20% levels used in this study) has been shown to enhance physiological performance and yield attributes in C_3_ crops without causing visible injury, its phytotoxic potential at higher concentrations or under different environmental conditions should be acknowledged. In this context, numerous studies indicate that foliar-applied methanol can act as an exogenous carbon source and may improve photosynthetic efficiency, water use, and growth under drought stress^45,46,88,90,93^. Recent studies indicate that methanol may enhance carbon availability and interact with endogenous metabolic pathways to support stress tolerance. However, experimental evidence from other plant systems suggests that the response to methanol is concentration-dependent and species-specific, with higher levels sometimes being ineffective or even detrimental to growth and yield^97^. Moreover, early mechanistic studies have shown that methanol applied to roots can cause phytotoxic damage, although foliar applications tend to be less harmful when used within a moderate range^98^. In the present experiment, no visible symptoms of phytotoxicity (such as leaf necrosis, chlorosis, or scorch) were observed across growth stages at 10% and 20% methanol, and all measured traits responded positively under drought stress, suggesting that these concentrations were within a stimulatory rather than toxic range. Nevertheless, the narrow threshold between beneficial and adverse methanol effects underscores the need for further research to establish crop-specific toxicity limits and optimal application windows in rapeseed and other crops.

Under abiotic stresses, particularly drought conditions, plants typically experience enhanced ROS production, including superoxide and H_2_O_2_, which can intensify oxidative damage to membranes, proteins, and the photosynthetic apparatus^64,99–101^. In response to drought, a coordinated antioxidant defense network is activated, comprising enzymatic components such as SOD, CAT, APX, and POD, along with non‑enzymatic antioxidants including CAR, proline, and soluble metabolites. Activation of these antioxidant systems helps mitigate ROS-induced oxidative damage and maintain cellular homeostasis under water-deficit conditions^69,102,103^. The intensity and efficiency of these responses often vary among plants, reflecting differential stress tolerance capacities. Higher SOD and CAT activities in some plants suggest a more robust redox regulatory system, enabling tighter control of ROS homeostasis under drought. Such variability is likely linked to genetic differences affecting antioxidant enzyme expression, redox signaling pathways, osmolyte biosynthesis, membrane stability, and intracellular compartmentalization efficiency^74,104^. Plants exhibiting stronger antioxidant regulation generally maintain chlorophyll integrity, preserve membrane functionality, and sustain metabolic activity more effectively under water deficit^105,106^. Therefore, variation in antioxidant responses should be interpreted as an indicator of differential acclimation capacity rather than merely a biochemical consequence of stress exposure.

Compatible osmolytes, particularly soluble carbohydrates and proline, also accumulate under drought and foliar treatments, contributing to osmotic adjustment, membrane stabilization, and ROS detoxification^31,107,108^. Osmotic adjustment not only stabilizes cellular homeostasis but also interacts with antioxidant defenses to reduce ROS accumulation and maintain metabolic function^109,110^. As a result, osmotic regulation enhances cell expansion, plant survival, and sustained growth during drought^111,112^. SC, in particular, accumulate significantly under drought and function as key osmoprotectants. Their increased concentration is positively correlated with osmotic balance, cell turgor maintenance, membrane stabilization, and protein protection^108^. By reducing cellular water potential and retaining intracellular water, soluble sugars promote chloroplast stability, photosynthetic activity, and drought resistance^113,114^. Furthermore, antioxidant enzyme activities, including CAT, GPX, and APX, generally increase proportionally with stress intensity, supporting enzymatic ROS scavenging and maintaining cellular integrity^115–117^. ROS-scavenging enzymes such as SOD, POX, CAT, glutathione reductase, and APX form a frontline defense system, and their activities are central to drought tolerance mechanisms^111,118–120^. In the present study, elevated levels of CAT, glutathione reductase, peroxidase, and proline under varying IR treatment indicated a strong oxidative stress response in rapeseed, especially in the second year of the experiment. On the other hand, SA and methanol acted synergistically, simultaneously supporting enzymatic ROS detoxification and metabolic homeostasis, ensuring both structural and functional stability of leaves under drought stress. Ultimately, the physiological enhancements induced by SA and methanol translated into improved GY and OY traits.

Quantitative relationships between grain yield and physiological traits were further examined using correlation analysis (Tables 7 and 8). As shown in Table 7, in the first year, GY was strongly correlated with thousand‑grain weight (TGW, *r* = 0.97, *p* < 0.001) and silique number per plant (SPP, *r* = 0.83, *p* < 0.001), as well as moderately associated with plant height (PH, *r* = 0.77, *p* < 0.001) and total chlorophyll content (Chl T, *r* = 0.78, *p* < 0.001). Similar trends were observed in the second year (Table 8), where GY was highly correlated with TGW (*r* = 0.92, *p* < 0.001) and SPP (*r* = 0.80, *p* < 0.001), confirming the consistency of these relationships across growing seasons. These results indicate that TGW and SPP are the primary determinants of GY in rapeseed under both well‑watered and drought conditions. Other traits, such as PHt and Chl T, also contributed positively to yield, albeit to a lesser extent, highlighting the importance of maintaining both morphological and physiological performance for optimal productivity. The consistency of these quantitative relationships across seasons underscores that key physiological traits are robust predictors of yield response under varying moisture regimes.

Furthermore, the foliar application of SA and methanol significantly enhanced these key traits under drought conditions. These quantitative analyses support the conclusion that the positive effects of SA and methanol on grain yield are physiologically justified, confirming that enhanced physiological performance under foliar treatments translates into tangible yield gains. All correlation data are presented in Tables 7 and 8, and the most relevant relationships have now been explicitly highlighted in the text, addressing the Reviewer’s comment regarding the need for quantitative interpretation of physiological traits in relation to grain yield. Foliar application of SA at 200 mg L^− 1^ and 20% methanol significantly increased TGW and SPP, demonstrating that improvements in photosynthetic performance, water status, and osmotic adjustment under these treatments directly enhance yield and oil production. These results align with reports showing that drought reduces seed yield and OY via fewer pods, lower seed numbers, and reduced seed weight^121–123^, and that methanol-containing treatments can mitigate these reductions by sustaining photosynthetic efficiency and assimilate translocation^124–126^. Overall, the present study demonstrates that foliar application of SA and methanol effectively alleviates drought stress in rapeseed by improving morphological, physiological, and biochemical traits, enhancing ROS detoxification, maintaining chlorophyll content, and supporting yield-determining traits. This integrated physiological and biochemical framework provides a deeper mechanistic understanding of how foliar treatments influence stress tolerance, addressing the reviewer’s concern about strengthening the analytical interpretation. In summary, foliar SA and methanol represent practical strategies to maintain rapeseed productivity under drought by integrating physiological, biochemical, and yield-related improvements.

## Conclusion

Drought stress poses a major challenge to rapeseed production, particularly during sensitive growth stages such as flowering and grain filling, by disrupting physiological processes and limiting yield formation. The present study highlights that targeted foliar applications of SA and methanol can enhance plant resilience under water-limited conditions by improving water status, photosynthetic performance, and antioxidant defenses. These treatments not only mitigate the negative impacts of drought but also translate physiological improvements into more stable and productive crop performance. The findings emphasize the potential of SA and methanol as practical tools for sustaining rapeseed productivity in semi-arid regions, providing a conceptually robust strategy to promote crop tolerance against environmental stresses.

## Data Availability

The datasets generated, used, deployed, and analyzed during this study are not publicly accessible. However, they are available from the corresponding author upon adequate request.

## Abbreviations

APX: Ascorbate peroxidase
CAR: Carotenoids
CAT: Catalase
Chl: Chlorophyll
Chl T: Total chlorophyll
Ea: Error a
Eb: Error b
FW: Fresh weight
GPS: Grains per silique
GPX: Guaiacol peroxidase
GY: Grain yield
IR: Irrigation regimes
LP: Leaf proline
OP: Oil percentage
OY: Oil yield
PHt: Plant height
POX: Peroxidase
RWC: Relative water content
SA: Salicylic acid
SC: Soluble carbohydrate
SL: Silique length
SOD: Superoxide dismutase
SPP: Siliques per plant
TGW: 1000-grain weight

## Declarations

### Declaration of compliance

We confirm and ensure that the study follows all relevant guidelines and regulations and complies with the IUCN Policy Statement on Research Involving Species at Risk of Extinction and the Convention on the Trade in Endangered Species of Wild Fauna and Flora.
